# A redox‐reactive delivery system via neural stem cell nanoencapsulation enhances white matter regeneration in intracerebral hemorrhage mice

**DOI:** 10.1002/btm2.10451

**Published:** 2022-11-16

**Authors:** Xuejiao Lei, Quan Hu, Hongfei Ge, Xuyang Zhang, Xufang Ru, Yujie Chen, Rong Hu, Hua Feng, Jun Deng, Yan Huang, Wenyan Li

**Affiliations:** ^1^ Department of Neurosurgery Southwest Hospital, Third Military Medical University (Army Medical University) Chongqing China; ^2^ Department of Emergency Affiliated Hospital, Zunyi Medical University Zunyi Guizhou China; ^3^ Institute of Burn Research, State Key Lab of Trauma, Burn, and Combined Injury, Chongqing Key Laboratory for Disease Proteomics Southwest Hospital, Third Military Medical University (Army Medical University) Chongqing China; ^4^ Institute of Materia Medica and Department of Pharmaceutics College of Pharmacy, Third Military Medical University (Army Medical University) Chongqing China

**Keywords:** intracerebral hemorrhage, layer‐by‐layer assembly, neural differentiation, neural stem cells, ROS‐scavenging

## Abstract

Intracerebral hemorrhage (ICH) poses a great threat to human health because of its high mortality and morbidity. Neural stem cell (NSC) transplantation is promising for treating white matter injury following ICH to promote functional recovery. However, reactive oxygen species (ROS)‐induced NSC apoptosis and uncontrolled differentiation hindered the effectiveness of the therapy. Herein, we developed a single‐cell nanogel system by layer‐by‐layer (LbL) hydrogen bonding of gelatin and tannic acid (TA), which was modified with a boronic ester‐based compound linking triiodothyronine (T3). In vitro, NSCs in nanogel were protected from ROS‐induced apoptosis, with apoptotic signaling pathways downregulated. This process of ROS elimination by material shell synergistically triggered T3 release to induce NSC differentiation into oligodendrocytes. Furthermore, in animal studies, ICH mice receiving nanogels performed better in behavioral evaluation, neurological scaling, and open field tests. These animals exhibited enhanced differentiation of NSCs into oligodendrocytes and promoted white matter tract regeneration on Day 21 through activation of the αvβ3/PI3K/THRA pathway. Consequently, transplantation of LbL(T3) nanogels largely resolved two obstacles in NSC therapy synergistically: low survival and uncontrolled differentiation, enhancing white matter regeneration and behavioral performance of ICH mice. As expected, nanoencapsulation with synergistic effects would efficiently provide hosts with various biological benefits and minimize the difficulty in material fabrication, inspiring next‐generation material design for tackling complicated pathological conditions.

## 1 INTRODUCTION

Intracerebral hemorrhage (ICH) is a severe disease that comes with high mortality and morbidity, bringing heavy burdens to the family and society.^1^ White matter injury following ICH is typical and responsible for various functional disorders.^2^, ^3^ However, the most widely applied therapies are still hematoma evacuation and supporting care, with scarce progress achieved for effective treatment aimed at white matter regeneration. Cell transplantation therapy applying neural stem cells (NSCs) has drawn great attention in recent years. NSCs are regarded as potentially helpful since they are able to differentiate into neurons, oligodendrocytes, and astrocytes and regulate the microenvironment to repair damaged white matter in ICH. Although functional recovery of ICH animals receiving NSC grafts has been demonstrated by some researchers, the general outcome is not satisfactory and clinical translation is slow.

There are two major obstacles in NSC transplantation for ICH. One is the apoptosis of transplanted NSCs caused by reactive oxygen species (ROS). In ICH, the accumulation of ROS in lesion sites is generated by secondary injuries, including heme degradation, iron overload, inflammation, and so on. Unlike primary injury from hematoma compression, which can be alleviated by surgery, secondary injuries persist so that ROS continuously compromises transplanted NSCs.^4^ Another is uncontrolled differentiation in the ICH microenvironment. NSCs tend to differentiate into astrocytes and seldom into oligodendrocytes, which are indispensable participants in remyelination and white matter regeneration.^5^ To overcome these two problems, different techniques have been applied. For example, NSCs were cotransplanted with exogenous growth factors in transplantation.^6^ Some studies genetically modified NSCs to overexpress regulators to promote neuroprotection in ICH.^7^ Our previous studies have also established techniques such as nanoparticles and hydrogels to eliminate ROS for cell protection.^8^, ^9^, ^10^ However, these methods usually address a single problem, lack controllable delivery and possess biosafety concerns, thus requiring further modifications.^11^

Among innovative techniques for improving NSC therapy in recent years, single‐cell nanoencapsulation has attracted great attention. This strategy utilized various biomaterial shells to encapsulate single cells through electrostatic adsorption, gelation, hydrogen bonding, and so on, in a layer‐by‐layer (LbL) manner, which helps to control the shell constituents and physical–chemical properties.^12^ For example, George's group utilized cell membrane glycans as an interacting coating component via click‐chemistry.^13^ Gao et al. even fabricated an encapsulation shell with deoxyribonucleic acids to enable programmed nanocoating.^14^ In our previous research, we established cell nanoencapsulation via electrostatic adsorption with biocompatible materials to deliver growth factors and verified cell protection.^15^, ^16^ However, nanogels via electrostatic adsorption lack sufficient rigidity and stability, making them unsuitable for grafting in ICH, which confers a formidable microenvironment. In this case, hydrogen bonding of naturally derived gelatin and tannic acid (TA) will be more appropriate since their interaction is rigid and biocompatible. Meanwhile, as a well‐known ROS scavenger, TA is believed to improve the disordered microenvironment.^17^ Importantly, the ROS elimination process can also be utilized as a trigger for payload release synergistically, through a synthesized ROS‐responsive prodrug linker (4‐nitrophenyl 4‐(4,4,5,5‐tetramethyl‐1,3,2‐dioxaborolan‐2‐yl)benzyl carbonate [NBC]).^18^ Triiodothyronine (T3) was chosen as the payload since it is a potent inducer to differentiate NSCs into oligodendrocytes and is small in size, which facilitates conjugation with the NBC linker. Thus, NSC protection and differentiation induction could be hopefully achieved synergistically by NSC nanoencapsulation.

Herein, we report a nanogel system of NSCs constructed with ROS‐responsive materials with synergistic benefits in cell protection and differentiation induction following transplantation in ICH. Aiming at the two major concerns involved in NSC therapy in ICH, single‐cell nanoencapsulation with ROS‐responsive materials delivering T3 was established. NSCs in the nanogels were successfully protected from ROS‐induced apoptosis via ROS scavenging which synergistically triggered T3 release to induce NSC differentiation into oligodendrocytes. Furthermore, ICH mice receiving nanogel grafts performed better in behavioral evaluation, neurological scaling, and open field tests. These animals exhibited enhanced oligodendrogenesis and regenerated white matter tracts, through activation of the αvβ3/PI3K/THRA pathway. This study further extended the application of cell nanoencapsulation in treating nervous system diseases, which has rarely been reported. The conversion of the coating shell into a stimuli‐responsive reservoir for synergistic effects also provided a next‐generation strategy in cell encapsulation functionalization for various purposes.

## 2 RESULTS

### 2.1 Synthesis and characterization of NBC and NBC‐T3

**SCHEME 1:**
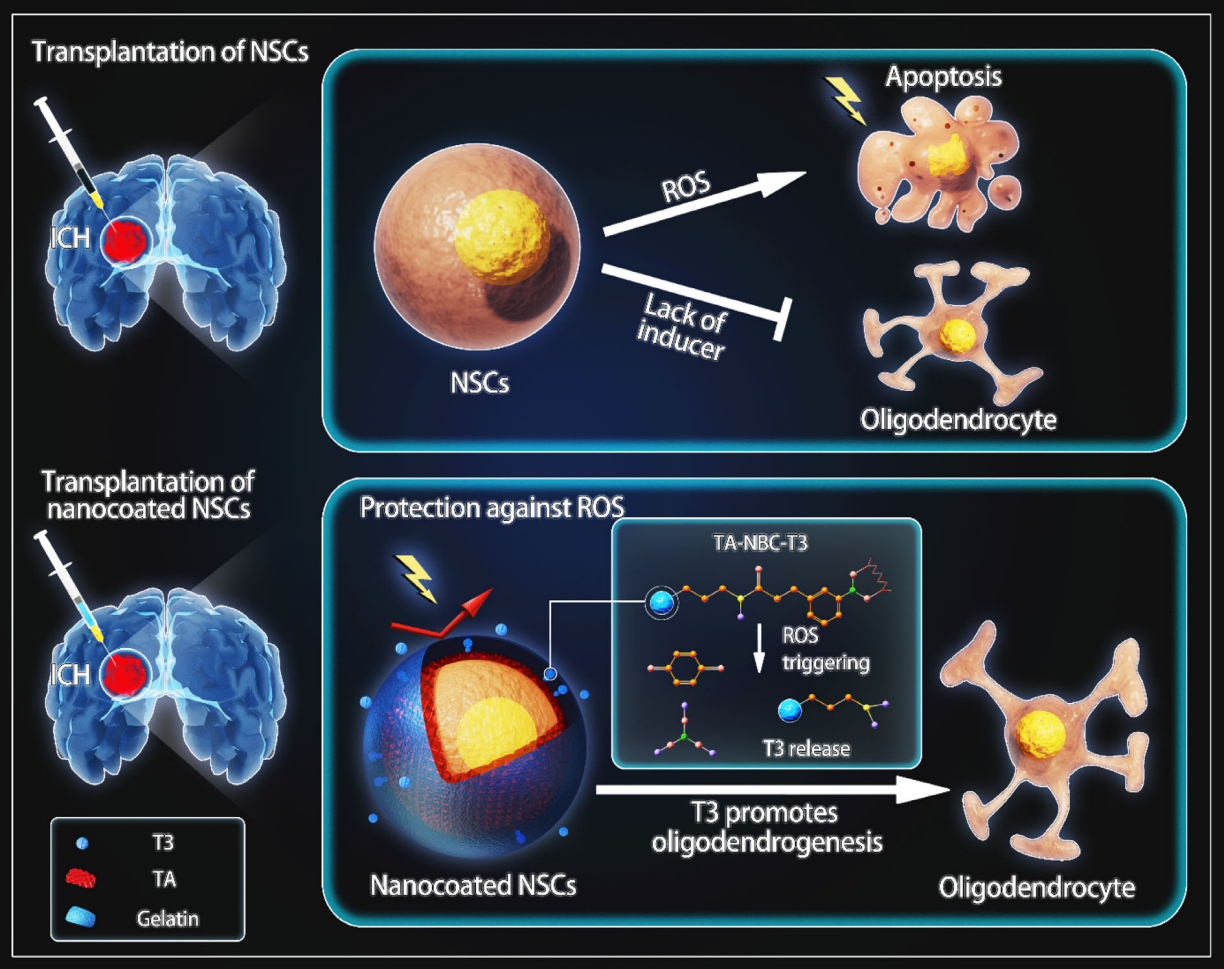
Design of ROS‐responsive T3 delivery through NSC nanoencapsulation in ICH cell therapy. ROS‐induced cell apoptosis and a lack of differentiation inducers are two major problems in NSC transplantation for ICH. By means of single‐cell nanoencapsulation with an ROS‐responsive T3 complex, apoptosis was attenuated, and oligodendrogenesis was promoted in transplantation therapy.

The obstacles of NSC transplantation and our strategy are depicted in Scheme 1. The first step was the fabrication of ROS‐responsive materials. Figure S1 demonstrates the synthetic processes of NBC and NBC‐T3. Characterization by NMR and mass spectrometry was significantly different between NBC and NBC‐T3 (Figure S2a–c). Interaction of TA with NBC‐T3 was shown in Figure S2d,e. The schematic picture in Figure S2f shows how TA‐NBC‐T3 was supposed to release T3 under ROS triggering. Following synthesis, the TNBS assay, which reflected the stability of the material by quantifying the free amino groups was performed on T3 and NBC‐T3 (Figure S2g). The amount of free amine groups contained in T3 was proportional to the amount of T3. In NBC‐T3, the reactive amine groups increased from 0 to 72 h due to degradation by oxidative sources from the environment. This indirectly proved the synthesis of T3 with NBC and the stability of NBC‐T3 over time. H_2_O_2_ at 0.2 mM, which is generally applied for to mimic pathological oxidative conditions in vitro was added to NBC‐T3 to test the triggered release of T3. The cumulative T3 release within 72 h was examined with a T3 ELISA kit, showing a ROS‐responsive and sustained release pattern (Figure S2h).

### 2.2 NSC nanogels were established via nanoencapsulation

**FIGURE 1:**
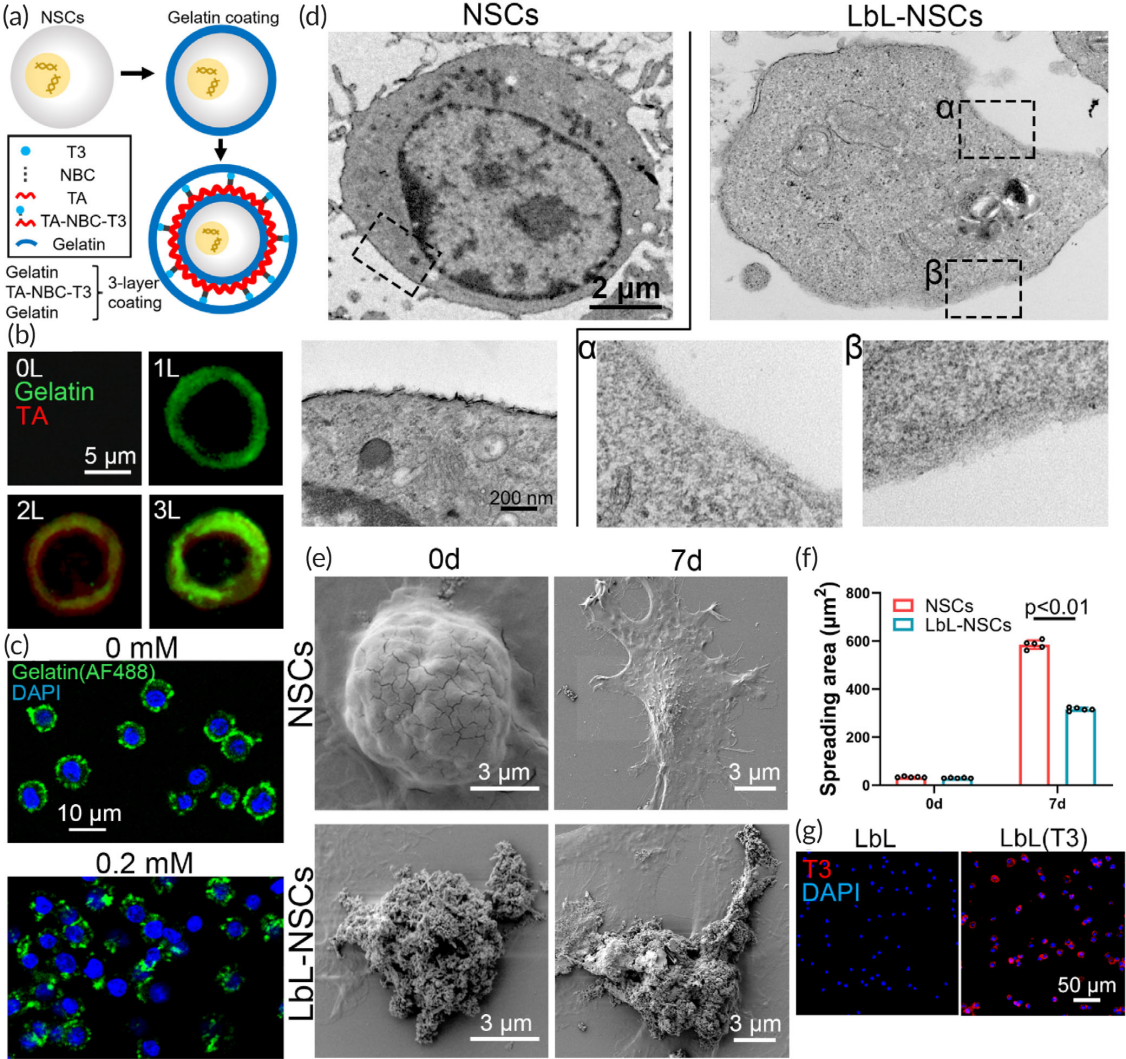
Characterization of layer‐by‐layer (LbL) nanogel. (a) Scheme of the process of LbL nanoencapsulation. (b) Neural stem cells (NSCs) without coating, NSCs coated with AF488‐labeled gelatin, AF488‐labeled gelatin/TA, and AF488‐labeled gelatin/TA/AF488‐labeled gelatin were observed with a confocal microscope. (c) LbL nanogels constructed with AF488‐labeled gelatin were treated with 0 or 0.2 mM H_2_O_2_ and examined with a confocal microscope. (d) NSCs and LbL nanogels (indicated as “LbL‐NSCs”) were examined by transmission electron microscopy (TEM). The images in the lower row are magnified views of the inserts in the upper row. (e) NSCs and nanogels seeded on cover slides were observed by scanning electron microscopy (SEM) on Days 0 and 7. The spreading area was calculated in (f) (*n* = 5). (g) Nanogel and T3‐incorporated nanogel were stained with anti‐T3.

The NSC nanogel was built via nanoencapsulation with gelatin as the inner protecting layer and outermost layer. NBC‐T3‐loaded TA was applied in the middle to form hydrogen bonds with the two gelatin layers (Figure 1a). In Figure 1b, NSCs were encapsulated with fluorescein‐labeled gelatin and TA from 0 to 3 layers and observed with a confocal microscope. Additionally, 1 day after the addition of 0.2 mM H_2_O_2_, the integrity of the coating shell was distinctly impaired, as shown in Figure 1c. Furthermore, untreated NSCs and NSC nanogel (labeled as LbL‐NSCs) were observed with TEM. In comparison with the cell surface of NSCs, materials coated on the cell membrane of LbL‐NSCs were apparent (Figure 1d). Cells from the NSC group and LbL‐NSC group were seeded and observed by SEM on Days 0 and 7. As shown in Figure 1e, the NSC nanogels presented materials on the surface of NSCs, and there was no material on the membranes of cells from the NSCs group. Additionally, due to the dynamic changes in the cytoskeleton, the spreading area of the cells gradually increased after grafting. Whether nanoencapsulation influences cell spreading was investigated. As calculated from Figure 1e, cells in the NSC and LbL‐NSC groups exhibited negligible differences in spreading area on Day 0. However, the spreading area in the LbL‐NSC group was significantly restricted on Day 7, with 316.05 ± 12.94 μm^2^ versus 582.14 ± 23.89 μm^2^ (Figure 1f). After nanoencapsulation with T3‐loaded materials, immunofluorescence staining with an antibody against T3 was performed to examine T3 expression on these nanogels. In Figure 1g, T3 expression was clearly observed on the cell surface, indicating successful encapsulation.

### 2.3 Nanogels protected NSCs from oxidative stress and direct oligodendrogenesis of NSCs in the ROS‐triggered pattern in vitro

**FIGURE 2:**
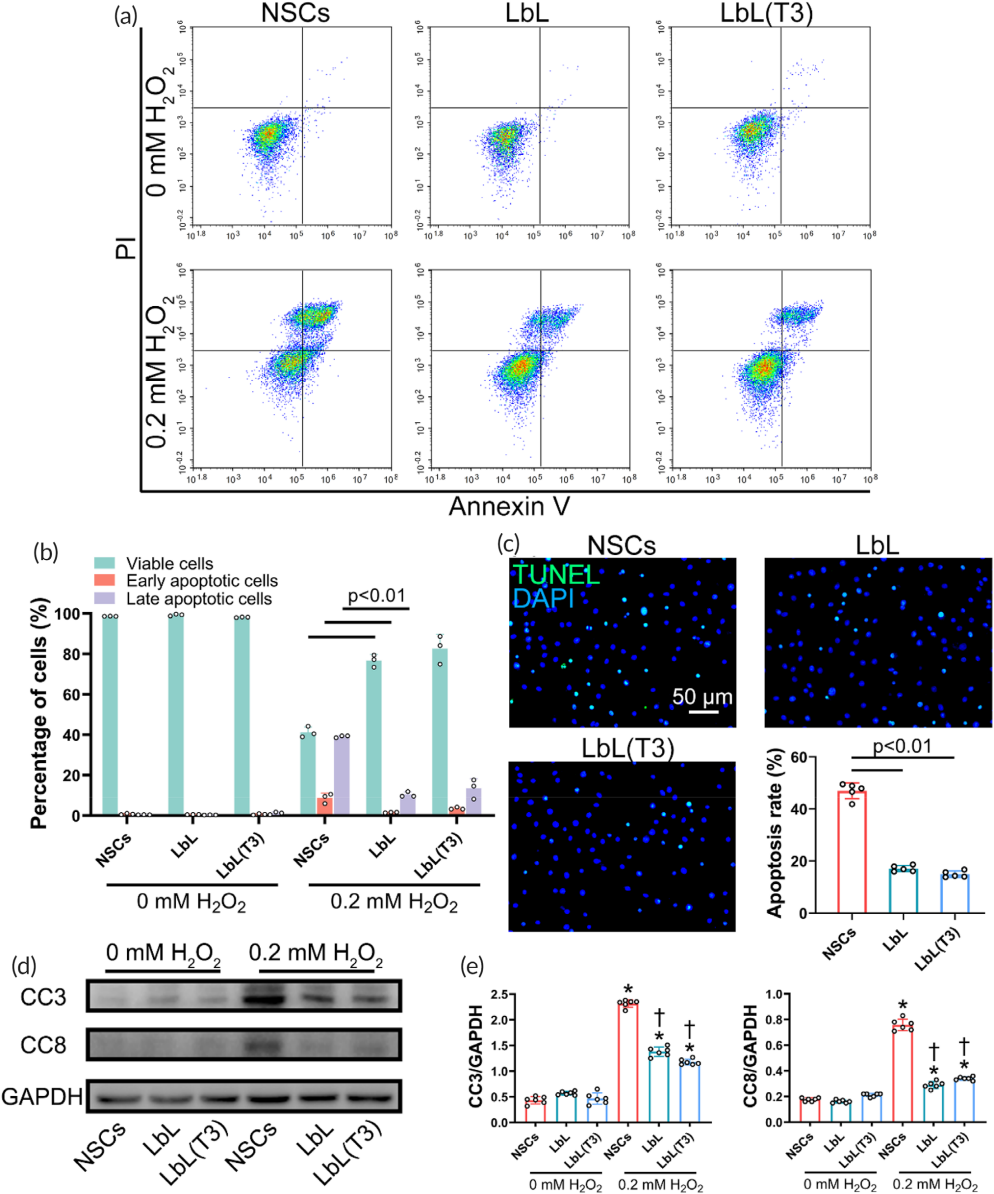
Protective effect of the nanogel against reactive oxygen species (ROS)‐induced apoptosis in vitro. (a) Flowcytometry was conducted to detect viable (Q2‐3), early apoptotic cells (Q2‐4), late apoptotic cells (Q2‐2), and necrotic cells (Q2‐1) after treatment with H_2_O_2_ at different concentrations for 1 h. Data analysis is shown in (b). (c) Neural stem cells (NSCs), LbL, and LbL(T3) were added with 0.2 mM H_2_O_2_ for 1 h and stained with a terminal deoxynucleotidyl transferase dUTP nick end labeling (TUNEL) apoptosis detection kit. Quantification was demonstrated (*n* = 6). (d) Western blot (WB) of cell samples from the NSC, LbL, and LbL(T3) groups was performed to determine the expression of the apoptosis pathways CC3 and CC8. (e) Quantification of WB results in (c). *p < 0.01 when the NSC group, LbL group, and LbL(T3) group (treated with 0.2 mM H_2_O_2_) versus NSC group, LbL group, and LbL(T3) group (treated with 0 mM H_2_O_2_), respectively; †*p* < 0.01 when LbL versus NSCs (treated with 0.2 mM H_2_O_2_) and LbL(T3) versus NSCs (treated with 0.2 mM H_2_O_2_) (*n* = 6)

NSCs, LbL nanogels, and LbL(T3) nanogels were treated with 0 and 0.2 mM H_2_O_2_ for 1 h. The protective effect of the nanogels on NSCs against ROS was verified by flowcytometry. As demonstrated in Figure 2a,b, both early and late apoptotic rates in the nanogel groups were significantly lower than those in the NSC group. At the same time, a TUNEL assay was conducted on these three groups. Figure 2c shows that the apoptosis rate in the nanogel groups was significantly lower than that in the NSC group. Additionally, protein samples were extracted from these cells to perform WB assays, exhibiting distinct activation of apoptosis pathways (Figure 2d). As quantified, the expression levels of CC3 and CC8 in the NSC groups following treatment with 0.2 mM H_2_O_2_ were greatly elevated. In addition, CC3 and CC8 levels in both nanogel groups were much lower than those in the NSC group, indicating attenuated apoptosis of NSCs following nanoencapsulation (Figure 2e).

**FIGURE 3:**
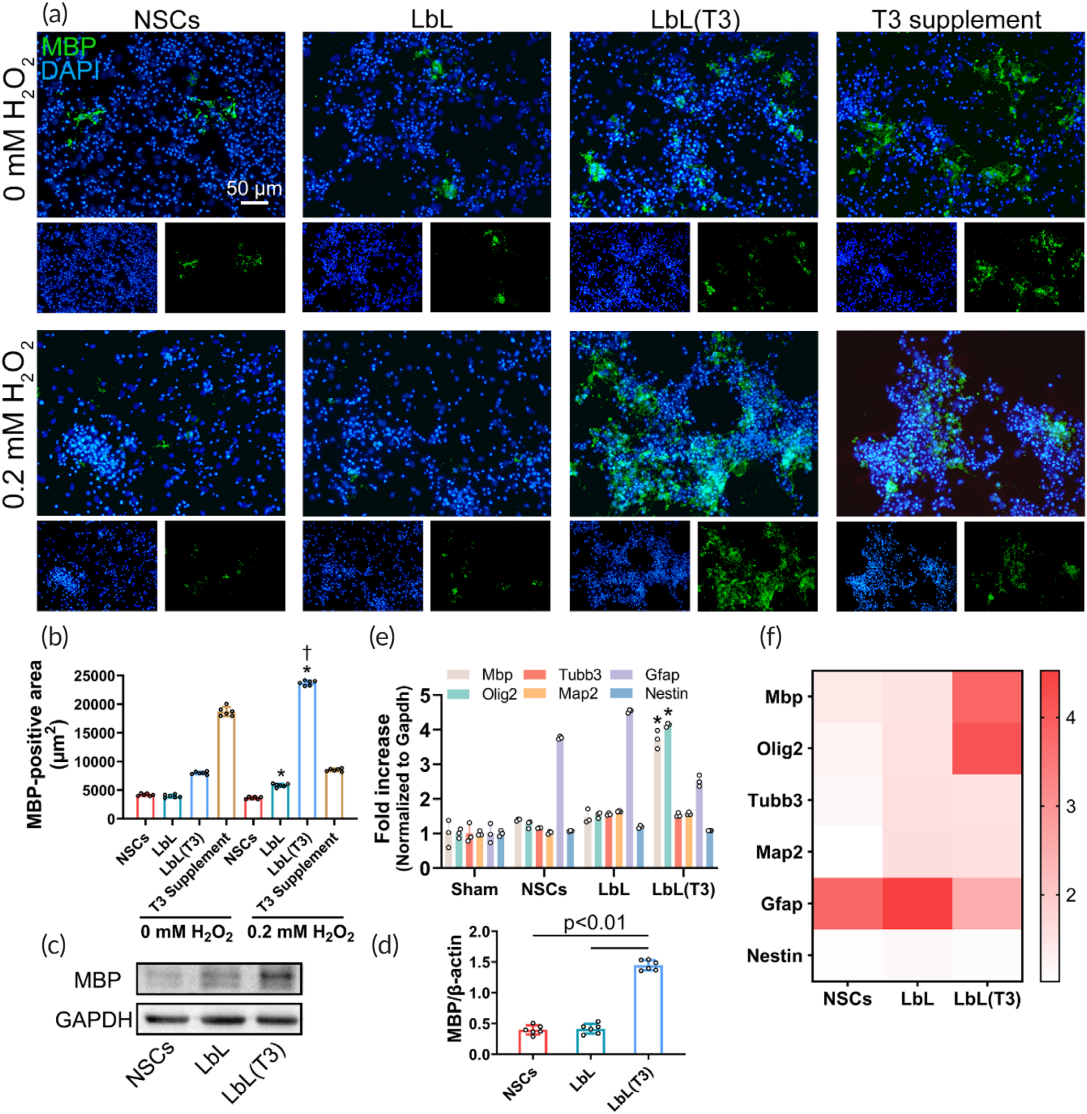
Oligodendrogenesis induction by LbL(T3) nanogel in vitro. (a) Neural stem cells (NSCs), layer‐by‐layer (LbL), LbL(T3) nanogels, and NSCs supplemented with 30 ng/ml were grafted onto slides and treated with different concentrations of H_2_O_2_. On Day 21, the cells were stained with anti‐MBP to demonstrate oligodendrogenesis. Split channels were displayed under each image. (b) Myelin basic protein (MBP)‐positive area obtained from (a). *p < 0.01 when LbL and LbL(T3) were treated with 0.2 mM H_2_O_2_ versus groups without H_2_O_2_ treatment; †*p* < 0.01 when LbL(T3) versus NSC, LbL, or T3 supplement groups with 0.2 mM H_2_O_2_ (*n* = 6). (c) Protein samples from NSC, LbL, and LbL(T3) groups with the addition of H_2_O_2_ on Day 21 were processed to undergo Western blot (WB). MBP expression was immunoblotted and then quantified in (d) (*n* = 6). (e) Real‐time qPCR of differentiation‐related genes from the NSC, LbL, and LbL(T3) groups with the addition of H_2_O_2_ on Day 21. Normalization was made against Gapdh expression (*n* = 3). *p < 0.01 for the expression of Olig2 and Mbp in the LbL (T3) group versus those in the NSC and LbL groups. (f) Heatmap of qPCR results from (e)

Oligodendrogenesis induction of the LbL(T3) nanogel was investigated. NSCs, LbL nanogel, and LbL(T3) nanogels were cultured on a 24‐well plate for 21 days. Immunofluorescence staining with anti‐MBP revealed the differentiation of oligodendrocytes (Figure 3a), which would produce myelin sheath in animal brain. The area of MBP‐positive cells could be quantified in Figure 3b, demonstrating that the area in the LbL(T3) group with H_2_O_2_ insult was 23730.23 ± 433.08 μm^2^, which was significantly increased compared with that in the LbL group and LbL(T3) group without ROS triggering. WB of the sample from the LbL(T3) group revealed higher expression of MBP than in the other groups (Figure 3c,d). Mbp and Olig2 were applied for oligodendrocytes, Tubb3 and Map2 for neurons and Gfap for astrocytes in qPCR (sequences of all the primers used are listed in Table 1). As quantified in Figure 3e, the expression levels of Olig2 in the LbL(T3) group were 3.3 and 2.6 times higher than those in the NSC and LbL groups; Mbp expression levels were 2.7 and 2.5 times higher than those in the NSC and LbL groups. A heatmap clearly demonstrates the variation in gene expression (Figure 3f).

**TABLE 1:** Primers used for RT‐qPCR

	Forward	Reverse
Mbp	5′‐TCACAGCGATCCAAGTACCTG‐3′	5′‐CCCCTGTCACCGCTAAAGAA‐3′
Olig2	5′‐GGCGGTGGCTTCAAGTCAT‐3′	5′‐CATGGCGATGTTGAGGTCG‐3′
Tubb3	5′‐CCCAGCGGCAACTATGTAGG‐3′	5′‐CCAGACCGAACACTGTCCA‐3′
Map2	5′‐GCCAGCCTCAGAACAAACAG‐3′	5′‐AAGGTCTTGGGAGGGAAGAAC‐3′
Gfap	5′‐CGGAGACGCATCACCTCTG‐3′	5’‐TGGAGGAGTCATTCGAGACAA‐3′
Nestin	5′‐CCCTGAAGTCGAGGAGCTG‐3′	5′‐CTGCTGCACCTCTAAGCGA‐3′
Src	5′‐GAACCCGAGAGGGACCTTC‐3′	5′‐GAGGCAGTAGGCACCTTTTGT‐3′
Thra	5′‐GGTCACCAGATGGAAAGCGAA‐3′	5′‐CCTTGTCCCCACACACGAC‐3′
Hif1α	5′‐TCTCGGCGAAGCAAAGAGTC‐3′	5′‐AGCCATCTAGGGCTTTCAGATAA‐3′
Mapk1	5′‐GGTTGTTCCCAAATGCTGACT‐3′	5′‐CAACTTCAATCCTCTTGTGAGGG‐3′
Thrb	5′‐GGACAAGCACCCATCGTGAAT‐3′	5′‐CTCTGGTAATTGCTGGTGTGAT‐3′
Gapdh	5′‐AGGTCGGTGTGAACGGATTTG‐3′	5′‐GGGGTCGTTGATGGCAACA‐3′

At the presence of 0.2 mM of H_2_O_2_, differentiation of cells in NSC and LbL(T3) groups on Days 7 and 21 was investigated. Figure S3a shows the ratios of differentiation into oligodendrocyte progenitor cells (OPCs) and oligodendrocytes on Day 7. Figure S3b shows the ratios into neurons and astrocytes. As analyzed in Figure S3c, it suggests that differentiation ratio of NSCs into oligodendrocytes was significantly higher in LbL(T3) group. Similarly in Figure S4 which exhibits the differentiation scenario on Day 21, promotion of oligodendrogenesis was further enhanced in LbL(T3) group as time passed by (Figure S4c).

### 2.4 Transplantation of NSC nanogels promoted neurologic functions in ICH mice

**FIGURE 4:**
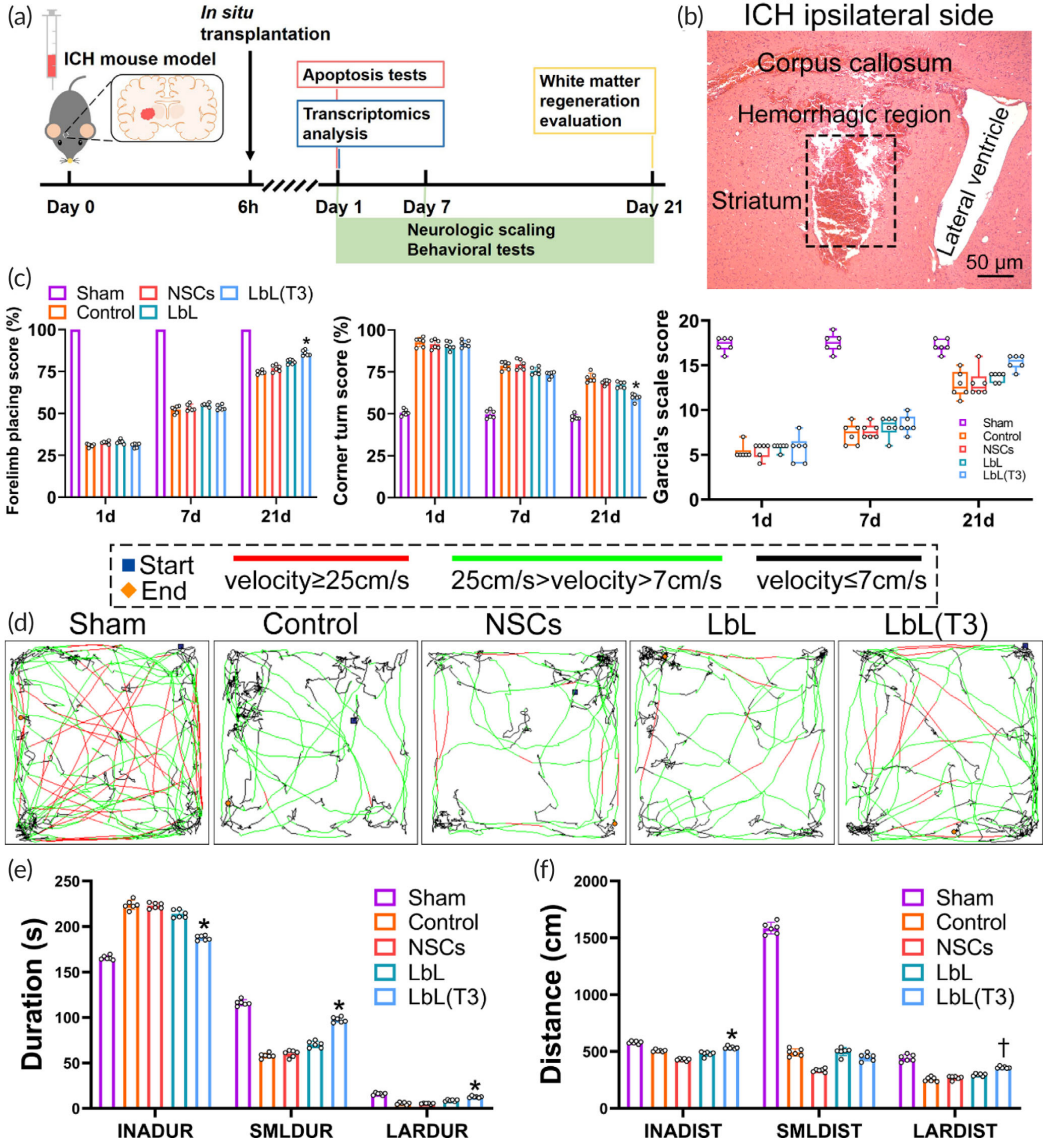
Functional recovery of intracerebral hemorrhage (ICH) mice in different groups. (a) Timeline of the research design. (b) H&E staining of brain slices in ICH. The framed region indicates the hematoma. (c) Mice from sham, control, neural stem cell (NSC), LbL, and LbL(T3) groups were assessed with a corner turn test on Days 1, 7 and 21. *p < 0.01 when LbL(T3) versus control, NSC or LbL groups (*n* = 6). Mice from different groups were assessed with forelimb placing test. *p < 0.01 when LbL (T3) versus control, NSC or LbL groups (*n* = 6). Mice from different groups were scored with Garcia's scale. *p < 0.05 when LbL (T3) versus control, NSC or LbL groups (*n* = 6). (d) Tracking paths of mice in open field tests. On Day 21, mice from the sham, control, NSC, LbL, and LbL(T3) groups were placed in the open field apparatus, and their movement paths within 5 min were tracked. Blue and orange squares indicate the starting and ending positions. Lines with different colors represent different speeds. (e) Durations (segmented as “inactivity,” “small,” and “large”) of different groups were calculated in locomotion tracking. *p < 0.05 when LbL (T3) versus control, NSC or LbL groups (*n* = 6). (f) Distances (segmented as “inactivity,” “small and “large”) were calculated. *p < 0.05 when LbL (T3) versus NSC; †*p* < 0.05 when LbL (T3) versus control, NSC or LbL groups (*n* = 6)

ICH mice receiving NSC grafts were evaluated for behavioral performance, white matter regeneration and mechanisms (Figure 4a). H&E staining demonstrated histologic characteristics of ICH lesions in the basal ganglia region (Figure 4b). Behavioral tests on mice such as corner turn and forelimb placing were applied following cell transplantation at different time points. From Figure 4c, the behavior scores were greatly promoted in the LbL(T3) group compared with the other groups on Day 21. The neurological function of mice at different time points was assessed by Garcia's scale. Although mice from all of these groups demonstrated recovery, mice receiving LbL(T3) grafts had higher Garcia's neurologic scores. Brain edema post‐ICH was investigated and the results at the time points are shown in Figure S3a. It clearly exhibited that edema in mice of the LbL(T3) group was better alleviated than in other groups on Day 21.

The open field behavioral test provided the locomotion and general activities of the animals, evaluated the functional recovery of the ICH mice from a comprehensive perspective. Mice from the sham, control, NSCs, LbL and LbL(T3) groups were placed in the open field test apparatus on Days 1 and 21. Their locomotion was recorded with a camera and the paths within 5 min were redrawn to exhibit the velocity segmentation and moving preference. Periods with velocities lower than 7 cm/s were defined as “inactivity” durations and the corresponding distances were defined as “inactivity” distances. Similarly, 7–25 cm/s was defined as “small,” and ≥25 cm/s was defined as “large.” Animal movement on Day 1 after ICH was generally impaired, as shown in Figure S5b–d. Figure 4d simply shows that mice from the control and NSC groups moved slower and preferred to stay in the corners, while mice receiving LbL and LbL(T3) nanogels showed faster locomotor activities and crossed the fields more, especially on Day 21. The quantification of the open field test is presented in Figure 4e,f. It was proven that mice from the LbL(T3) group had a significantly shorter inactivity duration, longer small duration and large duration on Day 21 compared with the other groups. The LbL(T3) group also exhibited a longer inactivity distance and large distance than the other groups. Simultaneously, the activity of mice during the period was segmented according to body motion at certain intervals. With the help of video recording analysis by the software, mouse activity was quantified by changes in pixels per frame (approximately 33.33 ms). A change less than 200 pix/frame was labeled “freezing,” a change between 200 and 500 was labeled “middle,” and a change larger than 500 was labeled “burst.” Figure S5e,f shows that mice from the LbL(T3) group exhibited a significantly shorter freezing duration, longer mid duration and longer burst duration.

### 2.5 Nanogel transplantation protected NSCs and enhanced white matter regeneration in ICH mice

**FIGURE 5:**
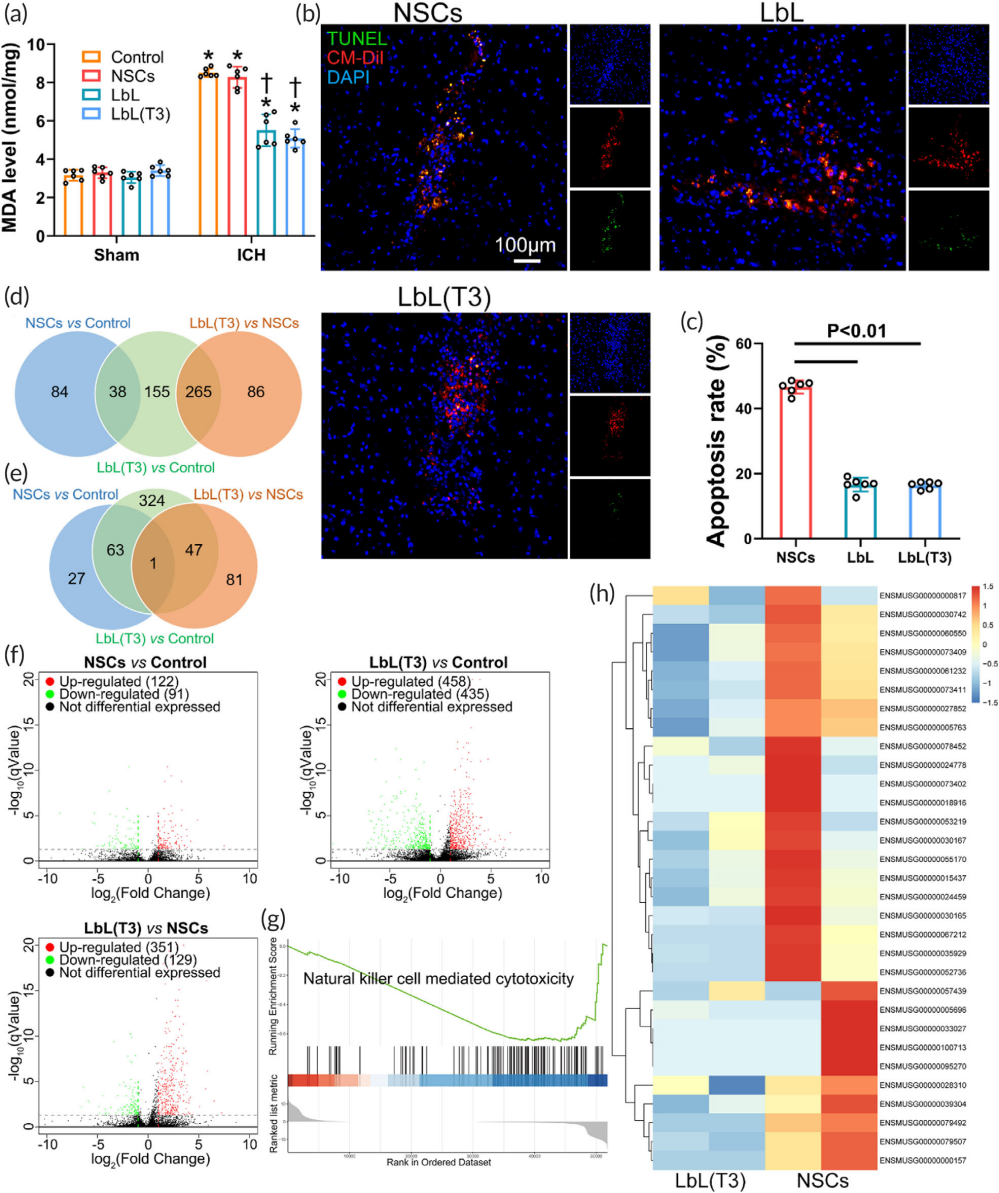
Protective effect of the nanogel on neural stem cells (NSCs) following transplantation. (a) Perihematoma tissue in the control, NSC, LbL, and LbL(T3) groups was collected to measure the peroxidation level with an malondialdehyde (MDA) assay. Tissue in the corresponding regions from the above groups was also collected from sham animals. *p < 0.01 when control (ICH) versus control (Sham), NSCs (ICH) versus NSCs (Sham), LbL (ICH) versus LbL (Sham) and LbL(T3) (ICH) versus LbL(T3) (Sham); †*p* < 0.01 when LbL versus control or NSCs (ICH animals); LbL(T3) versus control or NSCs (ICH animals) (*n* = 6). (b) Transplantation of CM‐Dil‐labeled NSCs, LbL nanogels, and LbL(T3) nanogels was performed in intracerebral hemorrhage (ICH) mice. After 24 h, brain samples were obtained to perform terminal deoxynucleotidyl transferase dUTP nick end labeling (TUNEL) apoptosis staining. The apoptosis rate was calculated in (c) (*n* = 6). (d) Venn diagram of co‐upregulated genes in different groups. (e) Venn diagram of co‐downregulated genes in different groups. (f) Volcano plots of gene expression in different groups. (g) Gene set enrichment analysis (GSEA) for the LbL(T3) and NSC groups. Natural killer cell‐mediated cytotoxicity was significantly suppressed in the LbL(T3) group. (h) Expression of genes from the natural killer cell‐mediated cytotoxicity pathway from the two groups

The protective effect of the nanogel in vivo is illustrated in Figure 5. Oxidative stress at the ICH site was reflected by MDA levels. Tissues surrounding the hematoma in the control group (without NSC transplantation), NSC group, LbL group and LbL(T3) group and tissues from the same sites in sham‐operated animals were dissected and homogenized for MDA quantification. As shown in Figure 5a, MDA levels were 3.42 ± 0.86, 3.61 ± 0.69, 3.14 ± 1.09, and 2.92 ± 0.82 nmol/mg in the sham control, NSC, LbL and LbL(T3) groups and 17.72 ± 1.62, 16.81 ± 1.23, 9.44 ± 0.93, and 9.20 ± 1.18 nmol/mg in the ICH control, NSC, LbL, and LbL(T3) groups, respectively. ROS obviously aggregated distinctly in all of these ICH groups, but the extent of aggregation was prominently decreased in the LbL and LbL (T3) groups. Furthermore, NSCs, LbL nanogels and LbL (T3) nanogels were grafted in situ 1 day after ICH. On Day 3, brain samples were sectioned and TUNEL apoptosis staining was conducted (Figure 5b). As calculated, the apoptosis rate of transplanted NSCs was 48.23% ± 3.25%, which was significantly higher than those in the LbL and LbL(T3) groups, which were 18.42% ± 2.12% and 17.11% ± 1.95%, respectively (Figure 5c).

Transcriptome analysis was performed on perihematomal tissue of ICH mice and ICH mice receiving NSCs and LbL(T3) nanogels. The Venn diagram in Figure 5d showed that 38 genes were co‐upregulated in NSCs versus control and LbL(T3) versus control; 265 genes were co‐upregulated in LbL(T3) versus control and LbL (T3) versus NSCs. In Figure 5e, 63 genes were co‐downregulated in NSCs versus control and LbL (T3) versus control; 47 genes were co‐downregulated in LbL(T3) versus control and LbL(T3) versus NSCs; 1 gene was co‐downregulated in NSCs versus Control and LbL (T3) versus NSCs. Volcano plots in Figure 5f demonstrated the number of up‐ and down‐regulated genes in different groups. Gene set enrichment analysis (GSEA) was applied to evaluate mRNA expression in perihematomal tissue of ICH mice receiving NSCs and LbL(T3) nanogels. Figure 5g demonstrates that natural killer cell‐mediated cytotoxicity was enriched and suppressed in the LbL(T3) group (enrichment score − 0.65, *p* = 0.01). The gene expression level associated with this gene set is shown in Figure 5h. In Figure S6, GSEA in perihematomal tissue of ICH and sham mice receiving NSCs was performed, showing activation of apoptosis‐related pathways in the ICH group. The results above suggested that LbL(T3) nanogels protected transplanted NSCs from apoptosis in ICH mice.

**FIGURE 6:**
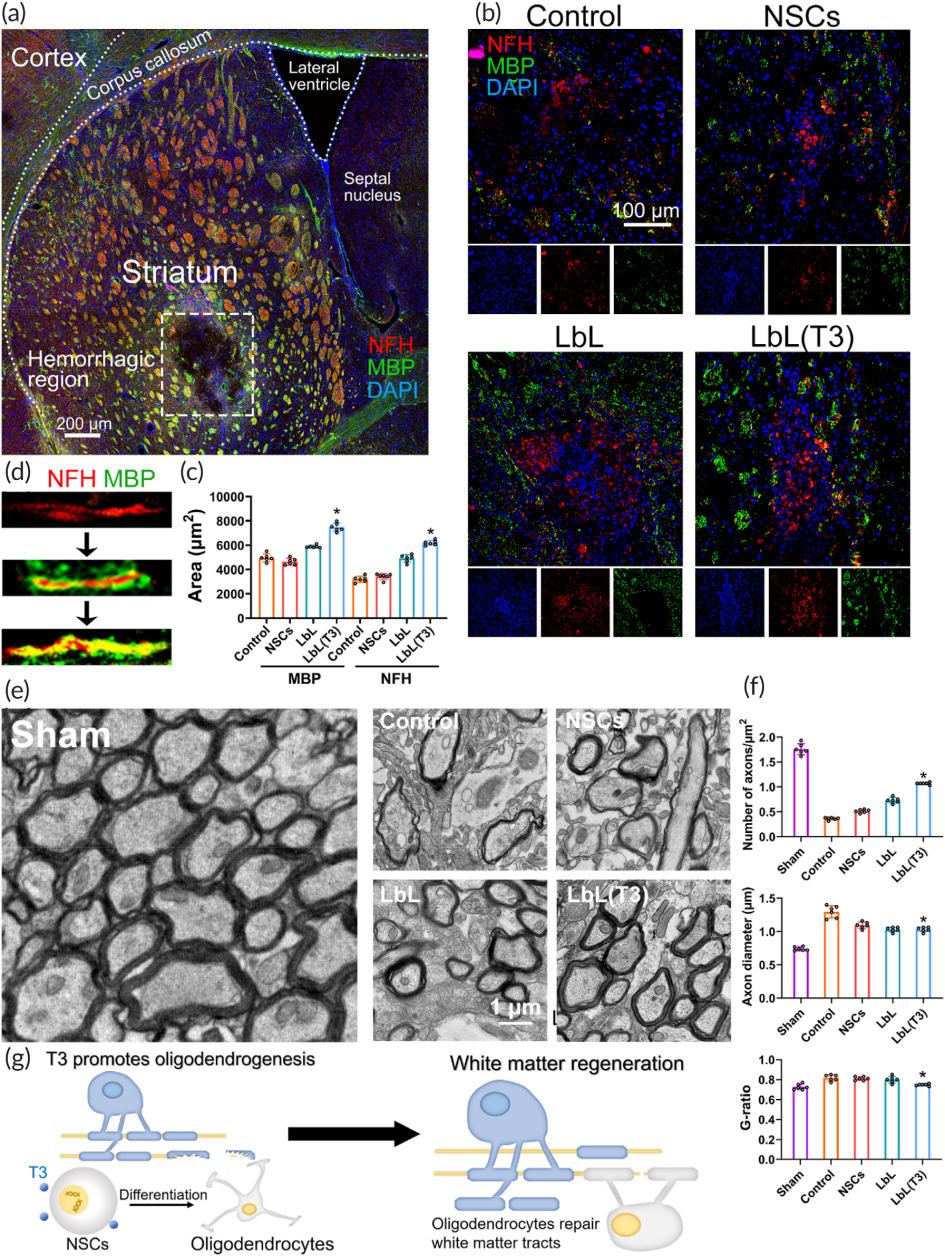
White matter injury and regeneration following neural stem cell (NSC) transplantation in intracerebral hemorrhage (ICH). (a) Immunostaining of brain slices from ICH mice. Perihematomal sites surrounding the framed area were further demonstrated in (b) for white matter regeneration. (b) On Day 21, brain sections of mice from the control, NSC, LbL, and LbL(T3) groups underwent fluorescence staining with anti‐MBP and anti‐NFH. Split channels were placed under each image. (c) MBP‐positive regions and NFH‐positive regions were quantified. *p < 0.01 when LbL(T3) versus control, NSC or LbL groups on Day 21 (*n* = 6). (d) Regeneration process of the white matter tract. (e) TEM images of perihematomal tissues in ICH from the control, NSC, LbL, and LbL(T3) groups on Day 21. Analysis of the axon number, diameter and G‐ratio is shown in (f). *p < 0.05 when LbL (T3) versus control, NSC or LbL groups (*n* = 6). (g) Scheme of oligodendrogenesis directed by released T3 and white matter regeneration by oligodendrocytes

Regeneration of white matter in mice receiving LbL(T3) nanogels was observed. On Days 3 and 21, mice without cell grafts, mice receiving NSCs, LbL nanogels and LbL(T3) nanogels were sacrificed to perform immunostaining on tissue sections, and the region of interest is framed in Figure 6a under low magnification. Figure S7 shows that the difference in the MBP‐positive area and axon growth was negligible on Day 3; however, on Day 21, oligodendrocyte and axon restoration were greatly enhanced in the LbL and LbL(T3) groups (Figure 6b,c). Figure 6d shows the myelination procedure.

TEM enabled us to obtain the ultra‐microstructure of white matter injury near the hematoma. On Day 21, perihematomal tissues from the sham, control, NSC, LbL, and LbL(T3) groups were obtained to undergo TEM examination (Figure 6e). As analyzed, there was a remarkable decrease in the number of axons in ICH animals, and significant recovery was found in the LbL(T3) group. Additionally, ICH also caused myelin edema, which was indicated by an enlarged axon diameter and G‐ratio. NSCs in the LbL(T3) nanogel demonstrated a significant reduction in these indicators (Figure 6f). The scheme in Figure 6g exhibits that T3 was released at the ICH site by ROS attack and then promoted the differentiation of NSCs into oligodendrocytes, which assisted white matter regeneration.

### 2.6 LbL(T3) nanogel promoted oligodendrogenesis via T3/PI3K/THRA signaling

**FIGURE 7:**
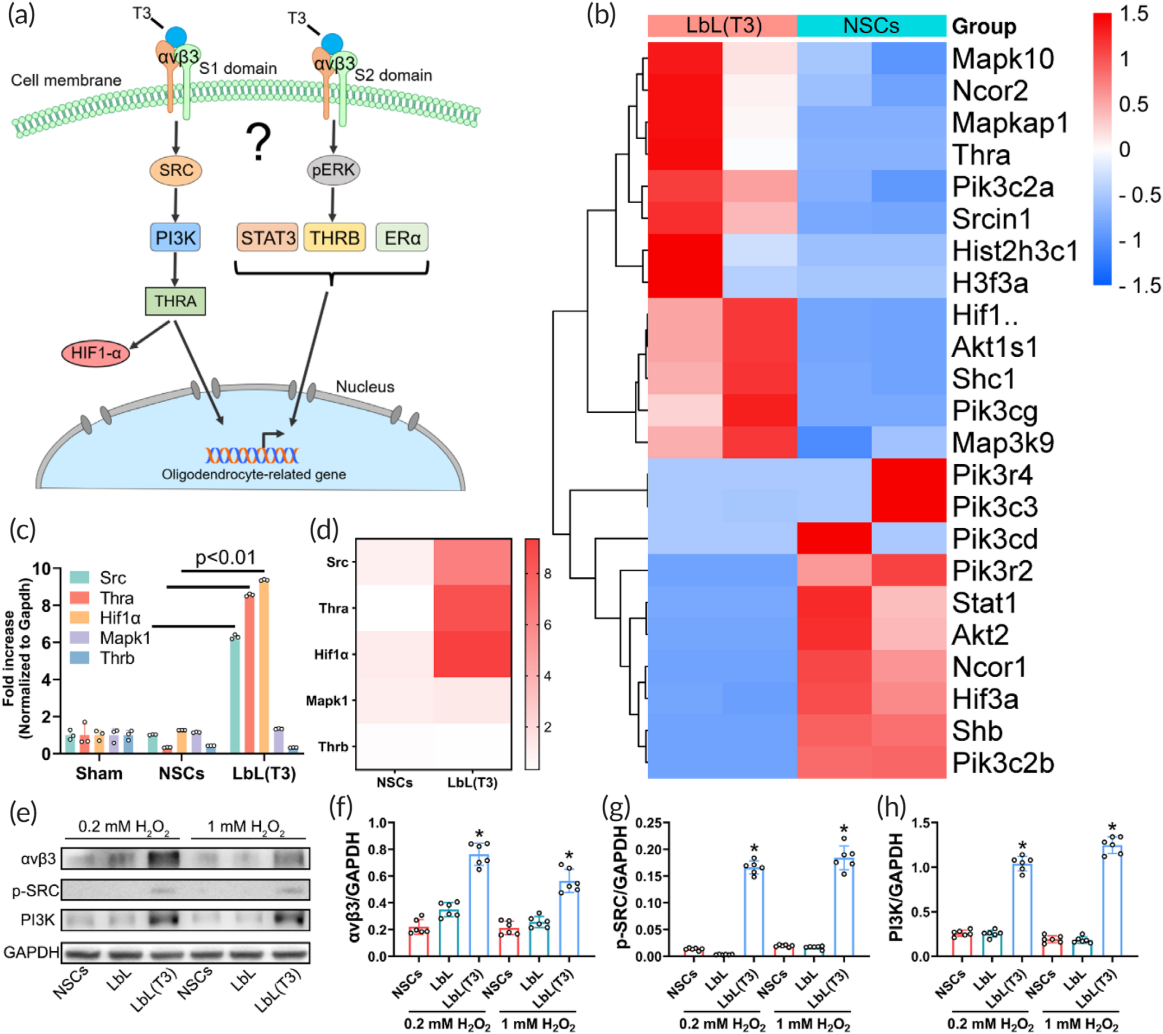
Signaling pathway involved in T3‐stimulated oligodendrogenesis. (a) Scheme of the pathways that T3 might activate in oligodendrogenesis. (b) Perihematoma tissues obtained from intracerebral hemorrhage (ICH) mice receiving neural stem cells (NSCs) and LbL(T3) were examined by transcriptome analysis. A heatmap of T3 pathway‐related gene expression is shown. (c) Real‐time qPCR of hallmark genes in the THRA and THRB pathways; the heatmap is depicted in (d). *p < 0.01 for the expression of Src, Thra, and Hif1α in the LbL(T3) group versus those in the NSC group (*n* = 3). (e) WB of cell samples from NSC, LbL, and LbL(T3) groups with treatments of 0 or 0.2 mM H_2_O_2_ and antibodies against αvβ3, p‐SRC and PI3K were used. (f)–(h) Quantification of WB in (e). *p < 0.01 when LbL(T3) versus NSCs or LbL (*n* = 6)

The oligodendrogenesis direction signaling was explored. T3 was presumed to interact with receptor αvβ3 through either domain S1 or S2 via the PI3K/THRA or pERK/THRB pathway (Figure 7a). Transcriptome analysis of mRNA expression in perihematomal tissue of the LbL(T3) nanogel and NSC groups was performed. Figure 7b shows the expression levels of related genes. The upregulation of the hallmark genes Thra, Hif1α, and Shc1 indicated the probability of T3/PI3K/THRA. Furthermore, gene expression evaluation with qPCR was again conducted to show the significant activation of Src, Thra and Hif1α in the LbL(T3) group compared with the NSC group (Figures 7c,d).

Cell samples from the NSC, LbL, and LbL(T3) groups following treatment with H_2_O_2_ in vitro underwent WB (Figure 7e). The expression of αvβ3, which acts as a T3 receptor, was greatly elevated in the LbL(T3) group compared to the NSC and LbL groups (Figure 7f). The protein levels of downstream SRC and PI3K were also increased in the LbL(T3) group (Figures 7g,h). Based on the fact that no difference was found when 0.2 or 1 mM was applied to the LbL(T3) nanogel, 0.2 mM, which mimicked the pathologic condition in hemorrhagic sites was enough to elicit T3 release. Taken together, T3 release triggered by ROS was supposed to induce oligodendrogenesis via T3/PI3K/THRA signaling, which was mediated by the αvβ3 S1 domain.

## 3 DISCUSSION

In this research, we have developed an ROS‐responsive T3 delivery nanogel on NSCs and demonstrated its role in promoting white matter injury in ICH mice, aiming at apoptosis alleviation and oligodendrogenesis induction following transplantation. This ROS‐responsive system took advantage of the synthesized compound NBC via a boronic linkage. The constructed LbL(T3) nanogel on NSCs exhibited protective effects against apoptosis. Following transplantation of nanogels, animals performed better in behavioral tests, and significant regeneration of white matter was verified. T3/PI3K/THRA signaling was suggested to be promoted to enhance the differentiation of NSCs into oligodendrocytes.

ROS, which are an acknowledged damaging factor, were leveraged as a trigger to release payloads.^10^ These ROS‐responsive materials are often based on boronic esters, mesoporous silicon, poly(thioketal), and so on.^19^, ^20^, ^21^ Boron‐derived compounds were attractive since they showed well‐defined properties of ROS‐sensing, the ability to link versatile payloads in aqueous solutions and degradability into innoxious phenols. In our study, NBC‐T3 was loaded on TA, and the composite material interacted with gelatin with stable hydrogen bonds. Without ROS attack, the payload would not be released so that T3 could be delivered precisely, avoiding burst release and minimizing drug waste.^22^ This was the first attempt to establish a smart material used in cell nanoencapsulation for transplantation therapy.

LbL nanoencapsulation could be established through different interactions.^23^ Our previous studies coated gelatin and alginate on NSCs to deliver insulin‐like growth factor‐1; however, the electrostatic force‐based structure was relatively weak and unstable. In 2010, Kozlovskaya et al. designed nanoencapsulation via hydrogen bonding with poly(N‐vinylpyrrolidone)/TA on yeasts, and hydrogen bond formation endowed a more rigid encapsulation shell than electrostatic adsorption and had less damage to mammalian cells than covalent bonding. Gelatin was then selected as the encapsulating pair because it exhibited ideal biocompatibility with NSCs and was reported to form hydrogen bonds with TA. Gelatin was nanocoated as an initial layer on the cell membrane via electrostatic force and then combined with TA through hydrogen bonding. Utilizing a protective gelatin layer was important since it prevented direct interactions between cell membrane ingredients with TA, which led to cell death, the reason for which was speculated to be impaired cell membrane components or the formation of an enclosed shell attached to the cell surface. In addition, the nanoencapsulation process was also optimized. Traditional LbL nanocoating costs approximately 5–10 min in material adsorption, while it took less than 1 min to form hydrogen bonds. This time‐saving procedure greatly reduced the time NSCs were exposed outside the chamber and better maintained the functions of NSCs.

With the aid of ROS‐responsive TA‐NBC‐T3, dual effects involving anti‐apoptosis and triggered release were achieved. On the one hand, ROS have been widely accepted as potent inducers of cell apoptosis. Targeting at ROS, the well‐known antioxidant TA was applied to help reduce ROS in focal sites and thus attenuate the apoptosis of nanoencapsulated NSCs.^24^, ^25^, ^26^ In comparison, the concentration of TA we applied was much lower so that the ROS scavenging efficiency was relatively lower. Cell apoptosis staining, transcriptome analysis, and WB demonstrated that at the ICH site, ROS‐induced apoptosis of NSCs was associated with cytotoxicity pathways. LbL(T3) nanogel transplantation effectively attenuated apoptosis, implying the benefit of ROS scavenging in controlling apoptosis, laying the foundation for white matter repair in the next steps. On the other hand, T3 release under ROS was demonstrated, and oligodendrogenesis by T3 was further verified. T3 is not only a hormone regulating the metabolism of the entire organism but also a stimulator of NSC differentiation into oligodendrocytes. In terms of the cell signaling, T3 was assumed to combine with the membrane receptor integrin αvβ3 and activate either the THRA or THRB pathway for oligodendrodenesis.^27^, ^28^ In this research, the delivered T3 was elucidated to interact with the αvβ3 S1 domain and promote downstream SRC/PI3K/THRA signaling, which then upregulated oligodendrocyte‐related phenotype expression. Oligodendrocytes helped remyelination and thus protected axons, improving white matter regeneration. However, successful oligodendrocyte induction could not guarantee white matter regeneration in vivo and requires evidence in animals.

Nevertheless, there are still limitations in our research. First, the mechanism of LbL nanogel disassembly requires exploration. It was speculated that the ROS‐responsive nanogel was able to react with ROS and might degrade, but the details should be clarified in the future because material degradation control is an important aspect of material modification for different applications. Second, the administration route should be further discussed. The reason why intracranial injection (in situ injection) was applied in our study was to mimic the situation in surgery. In most situations, ICH patients receive surgery only once, and thus it is rational to transplant NSCs while performing hematoma evacuation. Otherwise, there will be obvious ethical concerns about conducting a craniotomy exclusively for cell transplantation. In addition to intracranial injection, intravenous injection and transnasal delivery should be considered, and they call for additional engineering in targeting NSCs into lesion sites. Third, how T3 affects the differentiation mode in transplantation therapy is veiled. White matter repair needs certain number and ratio of differentiated neurons, oligodendrocytes, and astrocytes. From the in vitro study, released T3 promoted oligodendrogenesis and decreased neurogenesis in LbL(T3) group. Although white matter regeneration in ICH mice was evidenced to be promoted in LbL(T3) group, how the varied T3 amounts influence the differentiation mode and how to optimize the transplantation outcome by tuning the T3 component need further investigations.

## 4 CONCLUSION

We designed an ROS‐responsive T3 delivery system for NSC nanogel encapsulation and the synergistic benefits in NSC protection and differentiation induction following transplantation in ICH. The LbL nanogel was established via nanoencapsulation with gelatin/TA‐NBC‐T3 through hydrogen bonding. Protective effects on NSCs, ROS‐triggered T3 release and T3‐induced oligodendrogenesis were verified. Following intracranial transplantation of LbL(T3) nanogels, the functional recovery of the animals was greatly improved, and evident white matter regeneration was observed. Oligodendrogenesis of NSCs was significantly enhanced following the activation of the T3/PI3K/THRA pathway. This study further extended the therapeutic application of LbL nanoencapsulation in ICH and enlightened strategies of synergistic functioning for future material design.

## 5 MATERIALS AND METHOD

This part is provided in Supporting Information S1. All procedures which involve mice were approved by Laboratory Animal Welfare and Ethics Committee of the Third Military Medical University (No. AMUWEC20192038).

## AUTHOR CONTRIBUTIONS

**Xuejiao Lei:** Data curation (lead); methodology (lead). **Quan Hu:** Methodology (equal). **Hongfei Ge:** Data curation (equal). **Xuyang Zhang:** Methodology (supporting). **Xufang Ru:** Software (lead). **Yujie Chen:** Supervision (lead); validation (lead). **Rong Hu:** Investigation (lead). **Hua Feng:** Conceptualization (lead); resources (lead). **Jun Deng:** Formal analysis (lead); writing – review and editing (lead). **Yan Huang:** Methodology (equal); visualization (lead). **Wenyan Li:** Writing – original draft (lead).

## CONFLICT OF INTEREST

The authors declare no conflicts of interest.

### PEER REVIEW

The peer review history for this article is available at https://publons.com/publon/10.1002/btm2.10451.

## Supporting information


**Appendix S1:** Supporting Information

## Data Availability

The data that support the findings of this study are available from the corresponding author upon reasonable request.
