# Supplementary material for: A redox‐reactive delivery system via neural stem cell nanoencapsulation enhances white matter regeneration in intracerebral hemorrhage mice
Source: Bioeng Transl Med. 2022 Nov 16;8(2):e10451. doi: 10.1002/btm2.10451 (PMC10013746; doi:10.1002/btm2.10451)
Supplement: Supplementary file 1 — Appendix S1: Supporting Information [file BTM2-8-e10451-s001.docx]

Supporting Information

**A redox-reactive delivery system via neural stem cell nanoencapsulation enhances white matter regeneration in intracerebral hemorrhage mice**

Xuejiao Lei^1^, Quan Hu^2^, Hongfei Ge^1^, Xuyang Zhang^1^, Xufang Ru^1^, Yujie Chen^1^, Rong Hu^1^, Hua Feng^1^, Jun Deng^4^*, Yan Huang^3^*, Wenyan Li^1^*

^1^Department of Neurosurgery, Southwest Hospital, Third Military Medical University (Army Medical University), Chongqing 400038, China

^2^Department of Emergency, Affiliated Hospital, Zunyi Medical University, 149 Dalian Road, Zunyi, Guizhou 563003, China

^3^Institute of Materia Medica and Department of Pharmaceutics, College of Pharmacy, Third Military Medical University (Army Medical University), Chongqing 400038, China

^4^Institute of Burn Research, State Key Lab of Trauma, Burn, and Combined Injury, Chongqing Key Laboratory for Disease Proteomics, Southwest Hospital, Third Military Medical University (Army Medical University), Chongqing 400038, China

**MATERIALS AND METHOD**

Materials

Gelatin, tannic acid (TA), T3 and Peroxidation assay kit were purchased from Sigma-Aldrich. 4-(hydroxymethyl)phenylboronic acid pinacol ester, triethylamine, 4-nitrophenyl chloroformate and tetrahydrofuran were obtained from Macklin. Ham’s F-12 media, Dulbecco's Modified Eagle Medium (DMEM), B27 supplement, accutase, basic fibroblast growth factor (bFGF) and epidermal growth factor (EGF) were from Thermo Fisher (Gibco). Alexa Fluor 488 (AF488) N-hydroxysuccinimide (NHS) ester was obtained from Lumiprobe. Terminal deoxynucleotidyl transferase dUTP nick end labeling (TUNEL) assay kit, poly-L-lysine (PLL), bicinchoninic acid (BCA) kit and DAPI were from Beyotime. T3 enzyme-linked immunosorbent assay (ELISA) kit was bought from Abcam. Apoptosis detection kit was purchased from BioLegend. Anti-neurofilament heavy polypeptide (NFH) and myelin basic protein (MBP) were obtained from Abcam. Anti-αvβ3 was from Santa Cruz. Anti-phosphorylated extracellular signal-regulated kinase (pERK), anti-phosphoinositide 3-kinase (PI3K), anti-cleaved caspase-3 (CC3), and anti-cleaved caspase-8 (CC8) were purchased from Cell Signaling Technology. Anti-glyceraldehyde-3-phosphate dehydrogenase (GAPDH), secondary antibodies for immunofluorescence staining and horseradish peroxidase (HRP)-conjugated secondary antibodies were from ZSGM-BIO. C57BL/6 mice were purchased from SPF (Beijing) Biotechnology. MiniBEST Universal RNA Extraction Kit and reagent SYBR Premix Ex TaqII were obtained from TaKaRa.

*Synthesis of (4-nitrophenyl 4-(4,4,5,5-tetramethyl-1,3,2-dioxaborolan-2-yl)benzyl carbonate (NBC)*

4-nitrophenyl carbonochloridate (2.01g, 10 mmol) was added to a flask containing (4-(4,4,5,5-tetramethyl-1,3,2-dioxaborolan-2-yl)phenyl)methanol (2.34g, 10 mmol) and triethylamine (1.11 mL, 15 mmol) in dichloromethane (10 mL) at room temperature under Ar, and the reaction mixture was stirred at 0°C overnight. The yield of the NBC was 70% at a purity of 98%.

*Synthesis of NBC-T3*

The triiodothyronine (1.95 g, 3 mmol) and pyridine (1.1 mL, 4.5 mmol) dissolved in dichloromethane (10 mL) were added into a 250 mL three-necked flask under Ar, and then a solution of NBC (1.19 g, 3 mmol) in dichloromethane (10 mL) was added slowly at 0°C. After the reaction mixture was stirred at room temperature for 6 h, the resulting crude materials were purified by flash column chromatography on silica gel with MeOH/DCM (15:85, V/V) to give the desired products NBC-T3. The yield of the NBC was 40% at a purity of 95%.

*The preparation of TA-NBC-T3 fluorescence systems*

NBC-T3 (455 mg, 0.06 mmol) was added to a solution of [Tannic acid](javascript:;) (17 mg, 0.01 mmol) in phosphate-buffered saline (10 mL, pH 7.5). The mixture was filtered at room temperature after stewing at room temperature for 12 hours. The mixture was then freeze-dried to yield 322 mg of yellow solid.

*Culture of Neural Stem Cells (NSCs)*

As it was previously described[19]. E13.5 pregnant mice were obtained from Animal Research Center, Army Medical University. Followed by animal anesthetization with 5% isoflurane, tissues of abdominal region were spread apart. Furthermore, embryos from the sac were carefully collected, and then the brains of each embryo were taken. Cortices were carefully stripped off to be put into ice-cold Hank's Balanced Salt Solution (HBSS) and ground. After 3-times washing with HBSS, Accutase was added and the mixture were incubated under 37°C for 5 min. After discarding the digestive solution, NSC culture media (DMEM/F-12 plus 10 ng/ml bFGF and 10 ng/ml EGF) was added and the suspension was let to pass a cell strainer (mesh size: 25 μm). Finally, a T75 flask was used to culture the filtered cells and it was placed in an incubator with 5% CO_2_ and 100% humidity.

*NSC nanoencapsulation*

Based on previously established methodology, 2×10^6^ NSCs were firstly incubated in 1 ml 0.1% gelatin solution for 5 min with gentle shaking to coat the protecting layer. The suspension was then centrifuged at 800 rpm for 5 min and the supernatant was discarded. Next, NSCs underwent three times of washing with Dulbecco's phosphate-buffered saline (DPBS) and centrifuge. For the second layer, 0.3 ml 0.1% TA (or T3 loaded TA) was added and incubated for 1 min to form hydrogen bonds with gelatin. DPBS washing was again performed, and the third layer of 0.1% gelatin was finally coated.

*Intracerebral hemorrhage (ICH) model induction*

C57BL/6 Mice were anesthetized by 2.5% isoflurane inhalation and maintained with 1.5% isoflurane. Skull was then carefully exposed and a hole was drilled at the entry site of right basal ganglion which was located 0.2 mm anterior, 2.3 mm lateral and 3.5 mm deep to the bregma. Further, 20 μl of blood was collected from tail and slowly injected into the entry site on a stereotactic apparatus in 5 min. The syringe was retracted after another 5 min to prevent reflux. Finally, mice were placed in a thermostat at 37 ℃ and monitored till their recovery from anesthesia. For cell transplantation, 2×10^5^ NSCs or nanogels in 10 μl were injected 12 hours after ICH onset from the same hole, following aspiration of the blood for mimicking the hematoma removal.

*Scanning Electron Microscopy (SEM)*

SEM of cell samples was carried out as previously described. In general, NSCs from different groups were seeded on 0.01% PLL-precoated coverslips and 2.5% glutaraldehyde was added at different time points. Followed by fixation at 4 ℃ overnight and 3-times washing with DPBS, samples were dehydrated with graded ethanol and tert-butanol. Further, samples were sprayed with gold in vacuum and observed with a scanning electron microscope (Carl Zeiss). Spreading area of cells were quantified with ImageJ software.

*Transmission electron microscopy (TEM)*

For cell samples: NSCs from different groups were firstly fixed with 2.5% glutaraldehyde for 12 h. Samples were washed with DPBS for 5 min×3 times and then kept in 4 ℃ overnight. Post-fixation was processed with 1% OsO_4_ for 2 h. Following the fixation, cell samples were dehydrated with graded ethanol and embedded with epoxy resin. Further, samples were dried under 60 °C overnight and cut into ultrathin sections to mount. Finally, samples on copper grids were stained with 2% uranyl acetate and 1% lead citrate. A transmission electron microscope (JEOL, Japan) was operated to observe the prepared samples at room temperature.

For tissue samples: Animals from different groups were anesthetized and sacrificed. Brains were collected to immerse into 2.5% glutaraldehyde for 24 h. Tissues surrounding the hematoma were delicately dissected in 1×1×3 mm. Post fixation, dehydration, sectioning and observation were processed as described above.

*Preparation of AF488 labeled gelatin*

20 mg of gelatin was dissolved with 4 ml 0.1 M sodium bicarbonate buffer and 5 mg of AF488 NHS ester was dissolved in 1 ml of dimethylsulfoxide (DMSO). AF488 solution was then slowly added to gelatin solution while stirring. The mixture was maintained for 16 h in dark at room temperature. Finally, the solution was dialyzed for 3 days to get rid of the unbound AF488 agent and lyophilized.

*Flowcytometry*

Flowcytometry was performed to detect apoptosis with a FITC Annexin V/PI Apoptosis Kit according to the manufacturer’s instructions. Briefly, NSCs from different groups were grafted in 6-well plates and H_2_O_2_ of different concentrations was added. After 1 h, cells were rinsed with DPBS and incubated in working solutions. Finally, the samples were washed with DPBS and collected. An ACEA NovoFlow instrument (Omni Life Science) was used to detect the apoptotic, necrotic, and unaffected cells and the data was then analyzed with NovoExpress software (Omni Life Science).

*ELISA assay*

Standard, control, and experimental solutions were prepared in accordance with the manufacturer’s manual. Samples of 50 μl were added in the plate precoated with anti-T3 for 2 h at room temperature. Followed by 5 times of washing with the wash buffer, 100 μl conjugate solution was added to each well to incubate for 2 h. Further, substrate solution was incubated for 30 min in dark and 100 μl stop solution was mixed thoroughly with gently tapping. Finally, a microplate reader (Thermo varioskan flash) was applied to analyze the optical density at the wavelength of 450 nm.

*Brain water content*

On 1d, 7d and 21d post operation, brain water content of mice was examined to evaluate brain edema. As described previously[20], brains were removed from mice under anesthesia with intraperitoneal injection of 5% chloral hydrate and weighed immediately to obtain the wet weight. Furthermore, brain samples were kept in an oven at 80℃ for 48 h and then weighed to get the dry weight. Water Content (%) = [(Wet weight) -(Dry weight)]/ (Wet weight) ×100%.

*Garcia’s neurologic evaluation*

Neurologic functions of mice were evaluated with Garcia’s Neuroscore as described previously[21]. Specifically, spontaneous activity (3 minutes), side stroking, vibrissae touch, limb extension, forepaw outstretching and climbing (1 minute) of mice were assessed as 0 ~ 3, so that the scale system was ranging from 0 (maximal deficit) to 18 (intact). Mice were evaluated after surgery using the scale system on 1d, 7d and 21d.

*Behavioral tests*

Corner turn test: Animals were proceeded into a corner with the angle of 30 degrees. They would turn left or right to exit the corner. The process was repeated 10 times and the percentage of right turns was calculated.

Forelimb placing test: The test for each forelimb was conducted by brushing the vibrissae ipsilateral to that limb on a flat plane. Mice would place the forelimb rapidly on the plane once and the ratio of quick response was recorded.

*Open field tests*

Animals from different groups on day 1, day 7 and day 21 were placed in a box of 40 cm×40 cm and allowed to move freely for 5 min. The process was recorded with a camera installed overhead to analyze the movement paths, distances under different velocities, duration time under different velocities and activity quantization.

*Malondialdehyde (MDA) test*

Based on manufacturer’s manual, brain tissues adjacent to hematoma were dissected to be homogenized on ice, followed by MDA lysis buffer incubation. Samples were centrifuged at 13000 g to discard insoluble materials. Supernatant from the homogenized samples were taken for further analysis. Next, 600 μl of thiobarbituric acid was added to standard and experimental samples, incubating under 95 ℃ for 1 h. After cooling to room temperature, 200 μl of the reaction mixture was taken into a 96-well plate to detect the absorbance at the wavelength of 530 nm with a plate reader (Thermo Varioskan Flash).

*2,4,6-Trinitrobenzene Sulfonic Acid (TNBS) assay*

Different amounts of T3 protein were prepared in 0.1 M sodium bicarbonate at 0.1 mg/ml. Next, 250 μl of 0.01% TNBS was added to 500 μl of each sample, followed by incubation at 37 ℃ for 2 h. The reaction mixture was then added with 250 μl of 10% SDS and 125 μl of 1N HCl. Finally, the absorbance was examined at the wavelength of 530 nm with a plate reader (Thermo Varioskan Flash). The BSA protein was used as the standard sample.

*Immunofluorescence staining*

*For cell samples*: NSCs from different groups were grafted on cover slides and fixed in 4% paraformaldehyde (PFA) for 15 min. Cells were washed 3 times with DPBS for 5 min each and permeabilized with DPBS containing 0.1% Triton X-100 for 10 min. Next, 1% bovine serum albumin in PBST was added to block unspecific binding sites. Furthermore, primary antibodies diluted in blocking buffer were incubated under 4 °C overnight. After 3 times of washing with DPBS, fluorescent secondary antibodies were incubated for 1 h in dark at room temperature. Additionally, nuclei were stained with DAPI and washed with DPBS. Images were obtained with a confocal microscope (LSM780, Carl Zeiss). Primary antibodies: NFH (rabbit, 1:500) and MBP (mouse, 1:500). Secondary antibodies: AF594 chicken anti-rabbit (1:1500) and AF488 chicken anti-mouse (1:1500).

*For tissue samples*: Animals were anesthetized by intraperitoneal injection with 5% chloral hydrate and perfused with 4% PFA. Mice brains were collected for 25 μm-thick cryosection and the slices were then mounted on cover slides. Blocking, antibody incubation and imaging were processed as described above.

*TUNEL assay*

Brain cryosections were prepare as mentioned above. Furthermore, TdT buffer, fluorescence labeling buffer and reaction buffer were prepared according to the manufacturer’s instruction. After brain slices were washed with DPBS and permeabilized with PBST, these buffers were mixed to add 50 μl on each slice sample to incubate for 1 h in dark. Three-times washing with DPBS and DAPI nuclei staining were finally followed.

*Quantitative real-time polymerase chain reaction (qPCR)*

The total RNA of cell and brain samples were extracted with the  [RNA Extraction Kit](http://www.takara.com.cn/?action=Page&Plat=pdetail&newsid=1050&subclass=1). Next, cDNA synthesis was processed by RNA reverse transcription. According to manufacturer’s protocol, qPCR was performed with the SYBR Premix Ex TaqII by CFX96 System (Bio-Rad, USA). Gene expression was normalized using GAPDH and analyzed with the threshold (F=2^−ΔΔCt^). Sequences of primers were listed in Table 1.

*Western blot (WB)*

Different concentration of H_2_O_2_ were added in control group and nanoencapsulation groups. After 3 h of incubation, cells were washed with cold DPBS for 3 times and processed lysis with RIPA lysis buffer for 20 min. Protein quantitative detection was performed with a BCA kit, followed by loading buffer mixing and thermal denaturation. For electrophoresis, protein samples were loaded on sodium dodecyl sulfate-polyacrylamide gel electrophoresis (SDS-PAGE) gel in 20 μg/lane and immunoblotting was conducted on the polyvinylidene fluoride membranes. Primary antibodies: αvβ3 (mouse, 1:300), pERK (rabbit, 1:1000), PI3K (mouse, 1:1000), CC3 (rabbit, 1:1000), CC8 (mouse, 1:1000). Secondary antibodies: HRP-conjugated goat anti-rabbit (1:2000) and HRP-conjugated goat anti-mouse (1:2000). The blots were quantified with Image Studio software (LI-COR Biosciences).

*Transcriptome analysis*

RNA isolation. Total RNA was extracted using the TRIzol kit (Sangon, China) according to the manufacturer’s protocol, and treated with RNase-free DNase I to remove genomic DNA contamination. RNA integrity was evaluated with a 1.0% agarose gel. Thereafter, the quality and quantity of RNA were assessed using a NanoPhotometer® spectrophotometer (IMPLEN, USA) and an Agilent 2100 Bioanalyzer (Agilent Technologies, USA).

Library preparation and sequencing. A total amount of 2 μg RNA per sample was used as input material for the RNA sample preparations. Sequencing libraries were generated using Hieff NGS™ MaxUp Dual-mode mRNA Library Prep Kit for Illumina® following manufacturer’s recommendations and index codes were added to attribute sequences to each sample. Briefly, mRNA was purified from total RNA using poly-T oligo-attached magnetic beads. Fragmentation was carried out using divalent cations under elevated temperature in Hieff Frag/Prime buffer. First strand cDNA was synthesized using random hexamer primer and M-MuLV Reverse Transcriptase (RNase H-). Second strand cDNA synthesis was subsequently performed using DNA polymerase I and RNase H. Remaining overhangs were converted into blunt ends via exonuclease/polymerase activities. After adenylation of 3’ ends of DNA fragments, adaptor was ligated to prepare for library. In order to select cDNA fragments of preferentially 150~200 bp in length, the library fragments were purified and size-selected with Hieff NGS™ DNA Selection Beads. Then PCR was performed with Phusion High-Fidelity DNA polymerase, Universal PCR primers and Index (X) Primer. At last, PCR products were purified with Hieff NGS™ DNA Selection Beads（0.9×, Beads:DNA=1:1） and library quality was assessed on the Agilent Bioanalyzer 2100 system. The libraries were then quantified and pooled. Paired-end sequencing of the library was performed on the HiSeq NovaSeq 6000 sequencers (Illumina, CA).

Data assessment and quality control. FastQC (version 0.11.2) was used for evaluating the quality of sequenced data. Raw reads were filtered by Trimmomatic (version 0.36) according to several steps: 1) Removing adaptor sequence if reads contains; 2) Removing low quality bases from reads 3’ to 5’ (Q < 20); 3) Removing low quality bases from reads 5’ to 3’ (Q < 20); 4) Using a sliding window method to remove the base value less than 20 of reads tail (window size is 5 bp); 5) Removing reads with reads length less than 35 nt and its pairing reads. And the remaining clean data was used for further analysis.

Alignment with reference genome. Clean reads were mapped to the reference genome by HISAT2 (version 2.0) with default parameters. RSeQC (version 2.6.1) was used to statistics the alignment results. The homogeneity distribution and the genome structure were checked by Qualimap (version 2.2.1). BEDTools (version 2.26.0) was used to statistical analysis the gene coverage ratio.

Expression analysis. Gene expression values of the transcripts were computed by StringTie (version 1.3.3b). Principal Component Analysis (PCA) and Principal co-ordinates analysis (PCoA) were performed to reflect the distance and difference between samples. The TPM (Transcripts Per Million), eliminates the influence of gene lengths and sequencing discrepancies to enable direct comparison of gene expression between samples. DESeq2 (version 1.12.4) was used to determine differentially expressed genes (DEGs) between two samples. Genes were considered as significant differentially expressed if q-value <0.001 and |FoldChange| >2. When the normalized expression of a gene was zero between two samples, its expression value was adjusted to 0.01 (as 0 cannot be plotted on a log plot). If the normalized expression of a certain gene in two libraries was all lower than 1, further differential expression analysis was conducted without this gene. Gene expression differences were visualized by scatter plot, MA plot and volcano plot.

Functional analysis of differentially expressed genes. Functional enrichment analyses including Gene Ontology (GO) and KEGG was performed to identify which DEGs were significantly enriched in GO terms or metabolic pathways. Gene Ontology (GO) is an international standard classification system for gene function. DEGs are mapped to the GO terms (biological functions) in the database, the number of genes in every term is calculated, and a hypergeometric test is performed to identify significantly enriched GO terms in the gene list out of the background of the reference gene list. The Kyoto Encyclopedia of Genes and Genomes (KEGG) database is a public database of pathway data, KEGG pathway analysis identifies significantly enriched metabolic pathways or signal transduction pathways enriched in DEGs compared to a reference gene background, using the hypergeometric test. GO terms and KEGG pathway with false discovery rate (q-value) <0.05 were considered as significantly altered.

*Statistical analysis*

Means ± standard error of means was used to express the values. The error bars in charts expressed the standard error of means. One-way analysis of variance (ANOVA) was used to compare values from different groups in the study. The difference was considered to be significant when p value was less than 0.05.


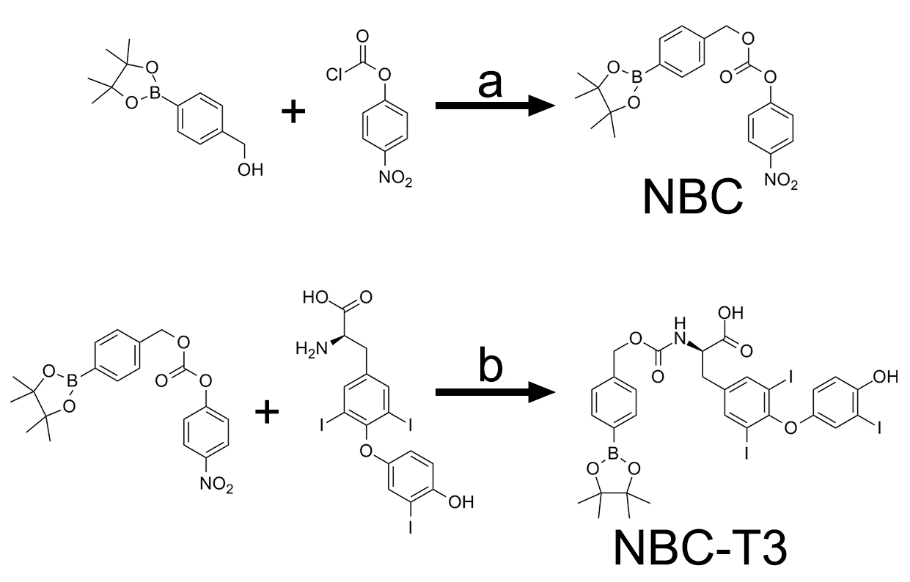


**Figure S1**. Schemes of the synthetic processes of NBC and NBC-T3. (a) TEA, Ar, 0 °C, overnight, 70%. (b) DIEA, Ar, 0 °C to room temperature, 6 h, 40%.


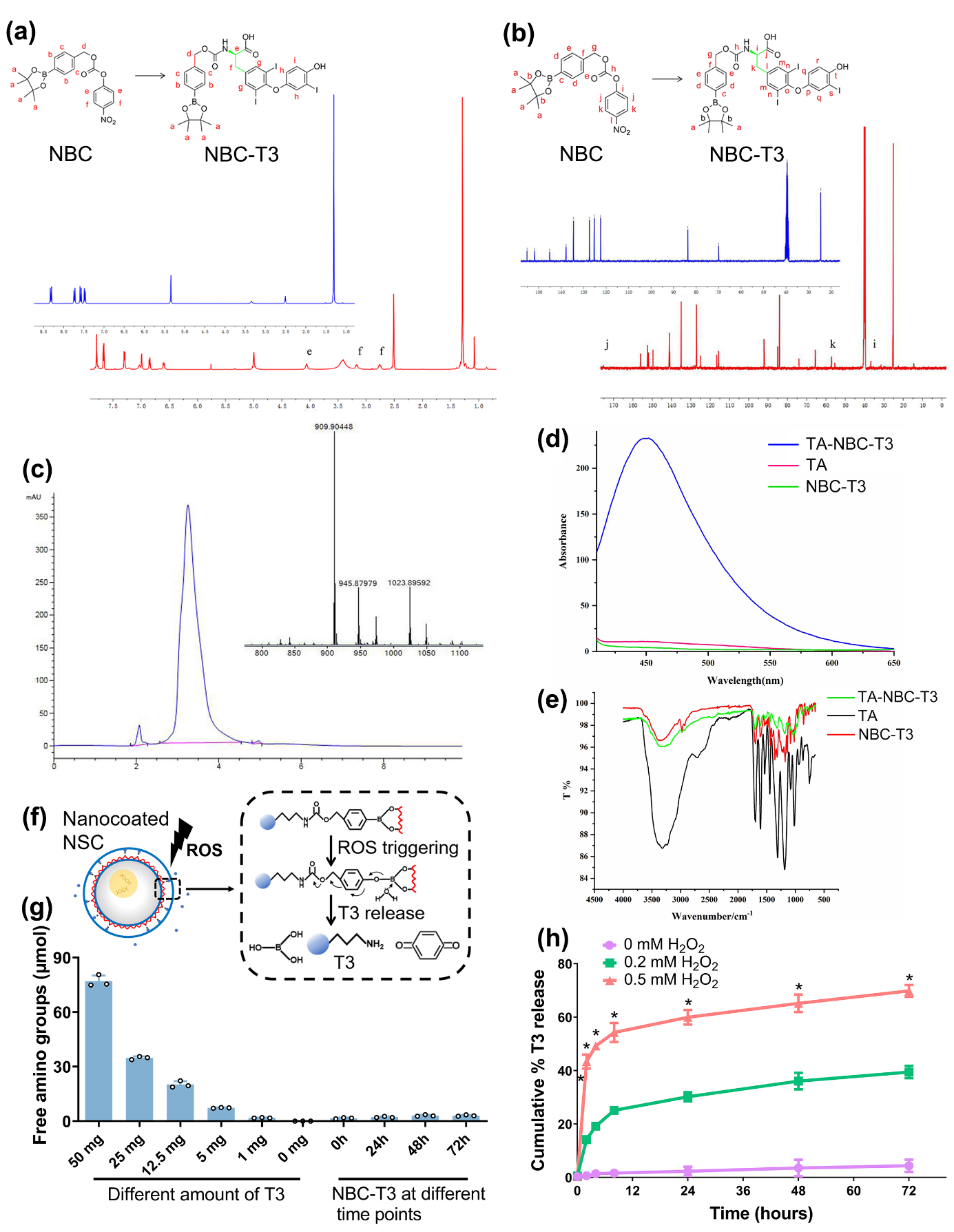
**Figure S2**. Synthesis of NBC and NBC-T3. (a) ^1^H NMR spectra of NBC (blue) and NBC-T3 (red) in deuterated DMSO. (b) ^13^C NMR spectra of NBC (blue) and NBC-T3 (red) in DMSO. (c) HRMS and HPLC of NBC-T3. (d) The fluorescence spectra of TA, NBC-T3 and TA-NBC-T3. (e) The FT-IR spectra of TA, NBC-T3 and TA-NBC-T3. (f) Scheme of how T3 was triggered to release by ROS. (g) TNBS exhibited reactive amino groups in different amounts of T3 and NBC-T3 compound at different time points. (h) T3 cumulative release of LbL(T3) nanogels under different concentrations of H_2_O_2_ was determined with a T3 ELISA kit at 0, 2, 4, 8, 24, 48 and 72 h. *: p<0.01 when LbL(T3) cells were treated with 0.5 mM H_2_O_2_ *vs.* those treated with 0 mM H_2_O_2_; when 0.2 mM H_2_O_2_ *vs.* 0 mM H_2_O_2_ (n=6).

NBC: ^1^H NMR (300 MHz, DMSO) δ 8.37 - 8.27 (m, 1H), 7.73 (d, J = 8.0 Hz, 1H), 7.64 - 7.53 (m, 1H), 7.48 (d, J = 8.0 Hz, 1H), 5.34 (s, 1H), 1.30 (s, 1H).

^13^C NMR (75 MHz, DMSO) δ 155.18, 151.83, 145.13, 137.83, 134.55, 127.42, 125.30, 122.47, 83.67, 70.00, 24.57.

MUDI-TOF m/z calcd. for C20H22BNO7 [M +] +399.2, found 399.2.

NBC-T3: ^1^H NMR (600 MHz, DMSO) δ 7.87 - 7.74 (m, 1H), 7.67 (d, J = 7.7 Hz, 1H), 7.30 (d, J = 7.5 Hz, 1H), 7.09 - 6.94 (m, 2H), 6.85 (d, J = 8.8 Hz, 1H), 6.67 - 6.51 (m, 1H), 5.20 - 4.73 (m, 1H), 4.06 (s, 1H), 3.17 (d, J = 10.6 Hz, 1H), 2.75 (dd, J = 18.0, 7.0 Hz, 1H), 1.29 (s, 12H).

^13^C NMR (151 MHz, DMSO) δ 156.03, 152.51, 151.97, 149.57, 141.26, 141.12, 140.95, 135.02, 127.12, 124.99, 116.58, 115.66, 92.08, 84.95, 84.11, 74.01, 65.51, 57.16, 36.78, 25.17.

MUDI-TOF m/z calcd. for C20H22BNO7 [M-H] + 909.9042, found 909.9042.


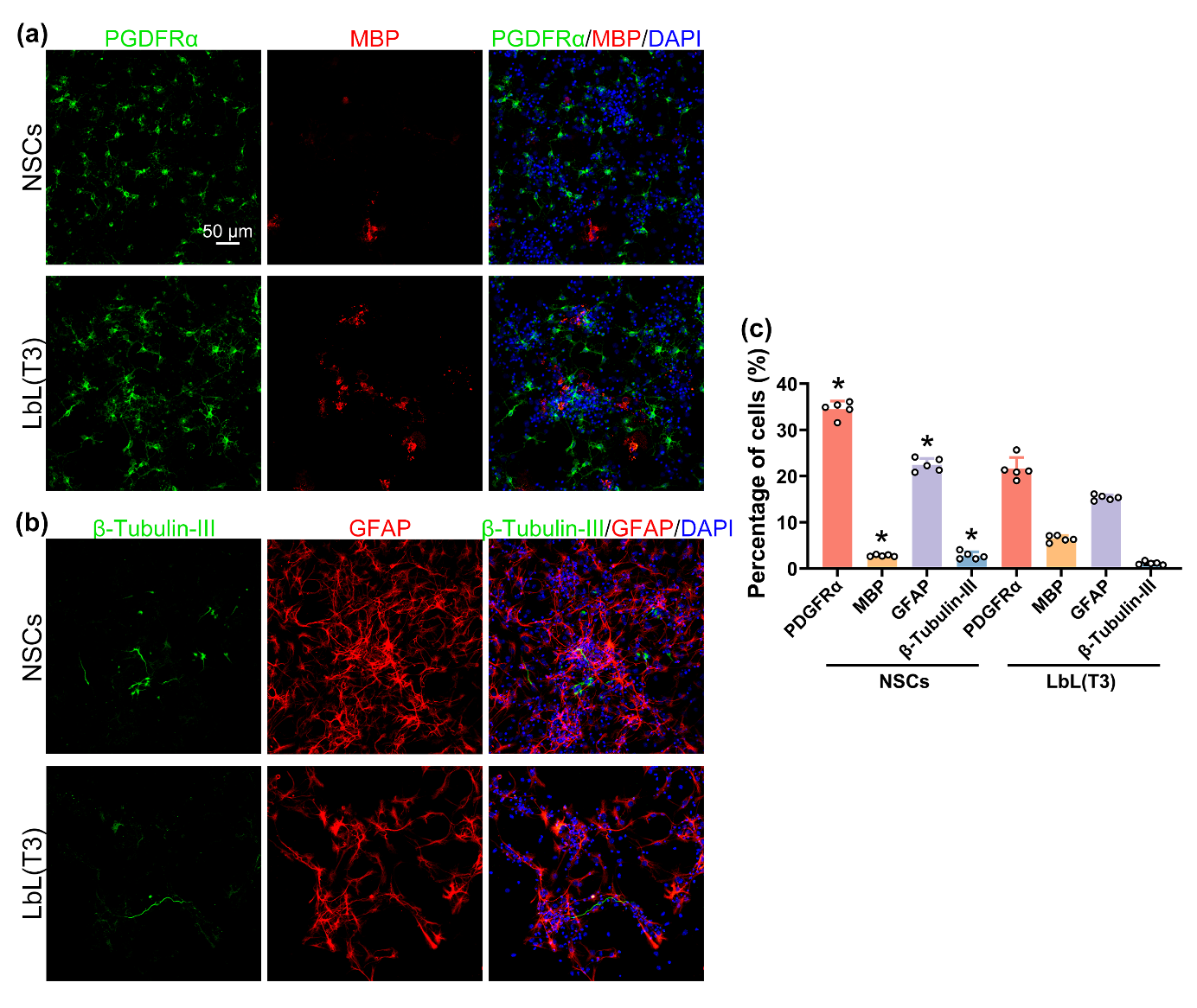
Figure S3. Cell differentiation in NSCs and LbL(T3) groups on day 7. NSCs and LbL(T3) nanogels were grafted onto slides and treated with different 0.2 mM H_2_O_2_. On day 7, the cell samples were processed with immunofluorescence staining. (a) Anti-PDGFRα+anti-MBP were applied to indicate oligodendrocyte progenitor cells and oligodendrocytes. (b) Anti-β-Tubulin-III+anti-GFAP were applied to indicate neurons and astrocytes. (c) The percentages of differentiated cells from (a) and (b) were quantified. *: p<0.01 when percentages of PDGFRα-positive cells, MBP-positive cells, GFAP-positive cells and β-Tubulin-III-positive cells in NSCs group *vs*. those in LbL(T3) group (n=5).


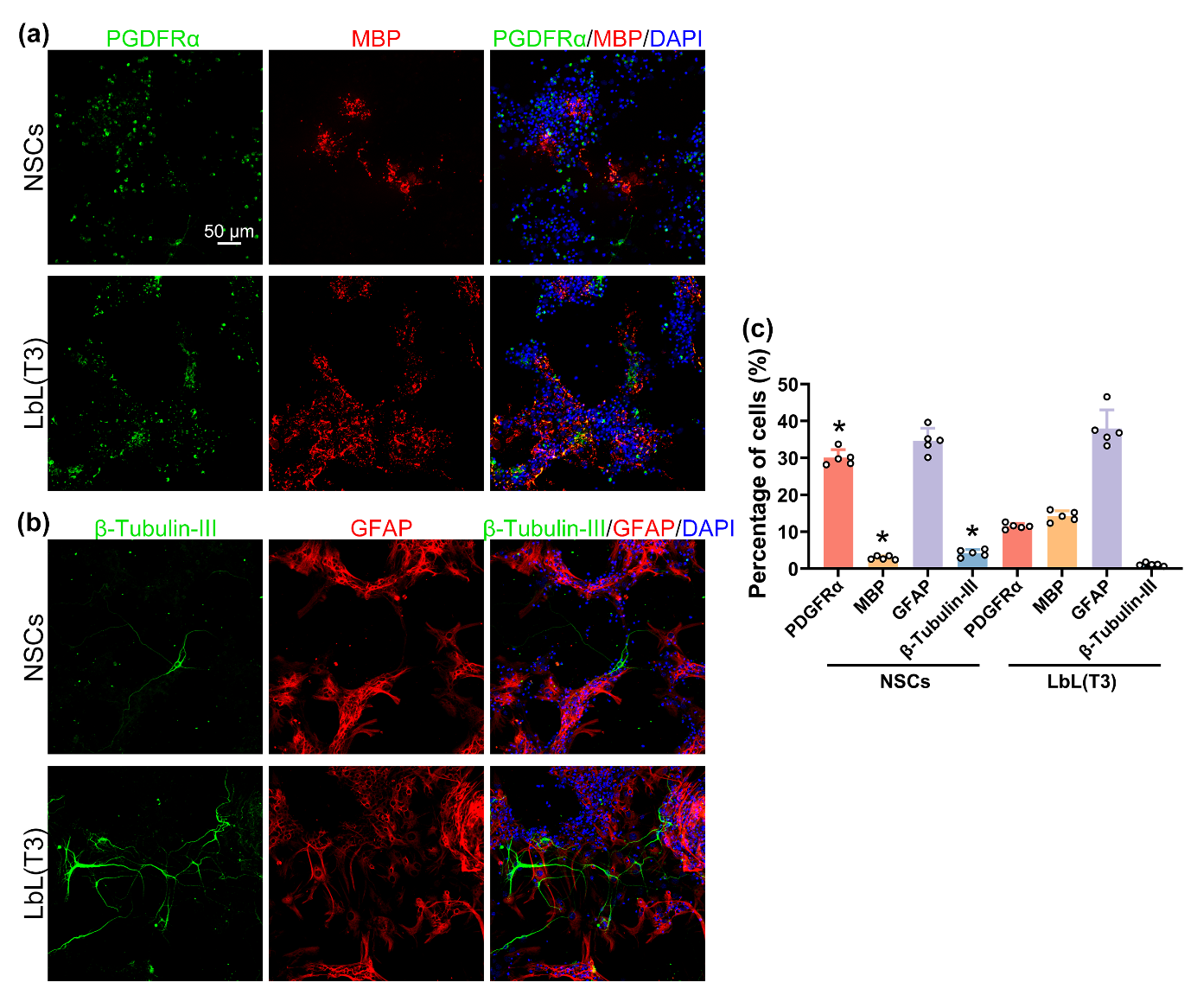
Figure S4. Cell differentiation in NSCs and LbL(T3) groups on day 21. NSCs and LbL(T3) nanogels were grafted onto slides and treated with different 0.2 mM H_2_O_2_. On day 21, the cell samples were processed with immunofluorescence staining. (a) Anti-PDGFRα+anti-MBP were applied to indicate oligodendrocyte progenitor cells and oligodendrocytes. (b) Anti-β-Tubulin-III+anti-GFAP were applied to indicate neurons and astrocytes. (c) The percentages of differentiated cells from (a) and (b) were quantified. *: p<0.01 when percentages of PDGFRα-positive cells, MBP-positive cells and β-Tubulin-III-positive cells in NSCs group *vs*. those in LbL(T3) group (n=5).


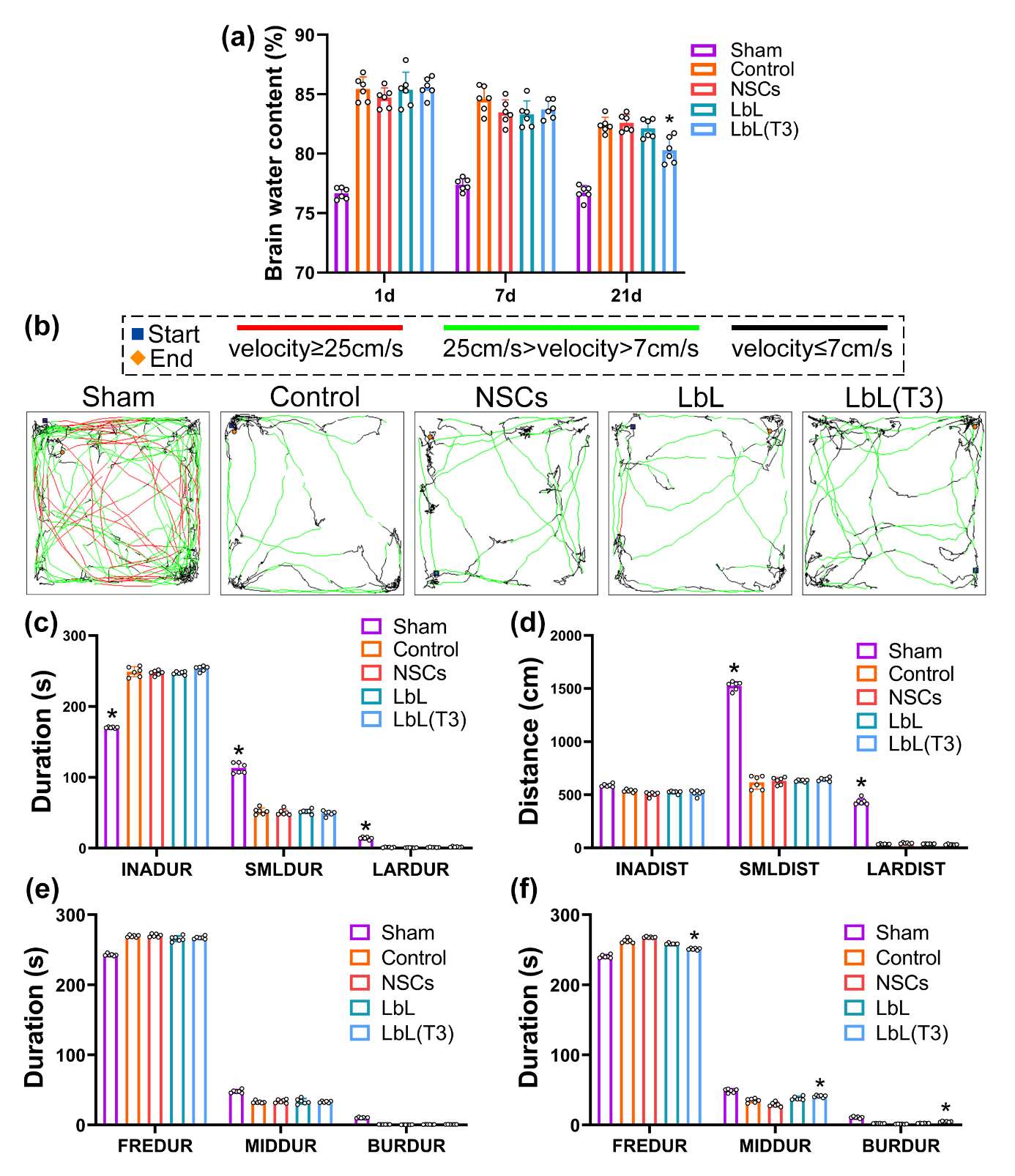
**Figure S5**. Brain edema, locomotion and activity of ICH mice in different groups. (a) Animal brains were obtained on day 1, day 7 and day 21, and the brain water content was evaluated. *: p<0.05 when LbL (T3) *vs.* control, NSC or LbL groups (n=6). (b) Tracking paths of mice in open field tests. On day 1, mice from the sham, control, NSC, LbL and LbL(T3) groups were placed in the open field apparatus, and their movement paths within 5 min were tracked. Blue and orange squares indicate the starting and ending positions. Lines with different colors represent for different speeds. (c) Durations (segmented as “inactivity”, “small” and “large”) of different groups were calculated in locomotion tracking. *: p<0.01 when sham *vs.* control, NSC, LbL or LbL(T3) groups (n=6). (d) Distances (segmented as “inactivity”, “small” and “large”) were calculated. *: p<0.01 when sham *vs.* control, NSCs, LbL or LbL(T3) groups (n=6). Durations (segmented as “freezing”, “mid” and “burst”) of different groups were calculated for activity characterization on day 1 (e) and day 21 (f). †: p<0.05 when LbL (T3) *vs.* control, NSC or LbL groups (n=6).


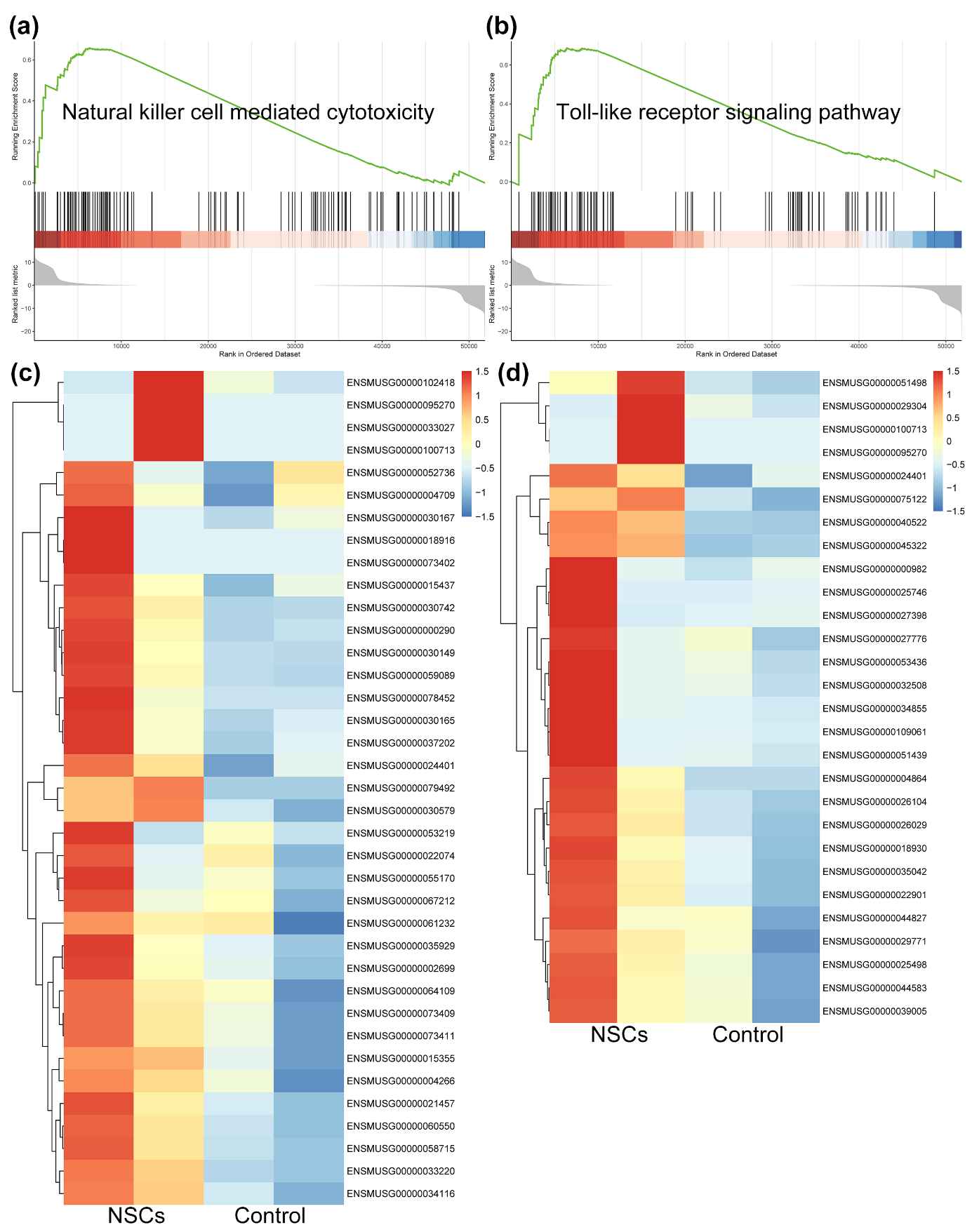


**Figure S6**. GSEA and gene expression. (a)-(b) GSEA for NSCs and Sham groups. Natural killer cell mediated cytotoxicity and Toll-like receptor signaling pathways were significantly activated in ICH mice that received NSCs compared to sham mice that received NSCs. (c)-(d) Expression of related genes from (a) and (b).


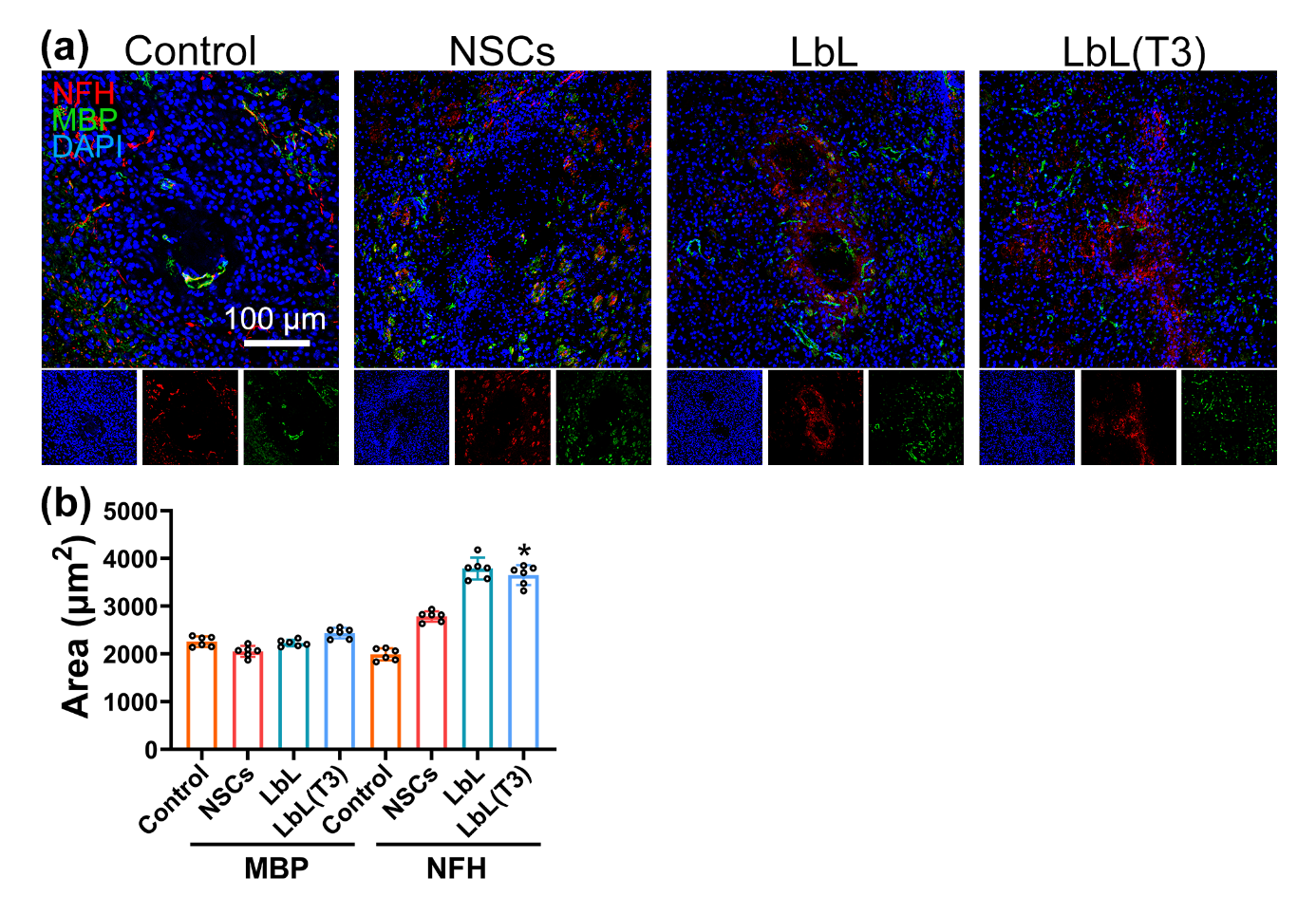
**Figure S7**. White matter injury following ICH. (a) On day 3, brain sections of mice from the control, NSC, LbL and LbL(T3) groups underwent fluorescence staining with anti-MBP and anti-NFH. Split channels were placed under each image. (b) MBP-positive regions and NFH-positive regions were quantified as the myelin area and axon area.
